# Mesopredatory fishes from the subtropical upwelling region off NW-Africa characterised by their parasite fauna

**DOI:** 10.7717/peerj.5339

**Published:** 2018-08-08

**Authors:** Katharina G. Alt, Thomas Kuhn, Julian Münster, Regina Klapper, Judith Kochmann, Sven Klimpel

**Affiliations:** Institute for Ecology, Evolution and Diversity, Johann Wolfgang Goethe University, Senckenberg Biodiversity and Climate Research Centre, Senckenberg Gesellschaft für Naturforschung, Frankfurt am Main, Germany

**Keywords:** Food-web, *Nealotus tripes*, Subtropical East-Atlantic, *Trichiurus lepturus*, Eastern boundary upwelling ecosystem, Canary Current

## Abstract

Eastern boundary upwelling provides the conditions for high marine productivity in the Canary Current System off NW-Africa. Despite its considerable importance to fisheries, knowledge on this marine ecosystem is only limited. Here, parasites were used as indicators to gain insight into the host ecology and food web of two pelagic fish species, the commercially important species *Trichiurus lepturus* Linnaeus, 1758, and *Nealotus tripes* Johnson, 1865*.* Fish specimens of *T. lepturus* (*n* = 104) and *N. tripes* (*n* = 91), sampled from the Canary Current System off the Senegalese coast and Cape Verde Islands, were examined, collecting data on their biometrics, diet and parasitisation. In this study, the first parasitological data on *N. tripes* are presented. *T. lepturus* mainly preyed on small pelagic Crustacea and the diet of *N. tripes* was dominated by small mesopelagic Teleostei. Both host species were infested by mostly generalist parasites. The parasite fauna of *T. lepturus* consisted of at least nine different species belonging to six taxonomic groups, with a less diverse fauna of ectoparasites and cestodes in comparison to studies in other coastal ecosystems (Brazil Current and Kuriosho Current). The zoonotic nematode *Anisakis pegreffii* occurred in 23% of the samples and could pose a risk regarding food safety. The parasite fauna of *N. tripes* was composed of at least thirteen species from seven different taxonomic groups. Its most common parasites were digenean ovigerous metacercariae, larval cestodes and a monogenean species (Diclidophoridae). The observed patterns of parasitisation in both host species indicate their trophic relationships and are typical for mesopredators from the subtropical epi- and mesopelagic. The parasite fauna, containing few dominant species with a high abundance, represents the typical species composition of an eastern boundary upwelling ecosystem.

## Introduction

Marine productivity is particularly high off the West African coast due to strong seasonal upwelling processes (Van Camp et al., 1991; Mbaye et al., 2015). The upwelling in the north-eastern Atlantic tropical upwelling system is mainly wind-driven and enhanced by the Gyre of Guinea in summer (Faye et al., 2015). This eastern boundary upwelling ecosystem (EBUE) is an important resource for fisheries (FAO fishing area 34, Eastern Central Atlantic), with 2.5% of annual catches worldwide originating from this region (FAO, 2012). Despite the economic relevance of West African fishery resources as well as records of overexploitation of small fish species, e.g., *Scomber japonicus* and *Engraulis encrasicolus*, data on commercially relevant fish species is scarce (FAO, 2009; Meissa, Gascuel & Rivot, 2013).

A fish species frequently encountered in the north-eastern Atlantic tropical upwelling region is the cutlassfish *Trichiurus lepturus* Linnaeus, 1758 (Trichiuridae). It inhabits the continental shelves to 350 m depths and can be found throughout temperate and tropical regions worldwide. Cutlassfish is a highly relevant commercial fishery resource with stable annual captures of 1.3 million tons since 2008 (Nakamura & Parin, 1993; FAO, 2014). As one of the ten most important species targeted by marine fisheries worldwide, its annual take is similar to other commercially important species, e.g., yellowfin tuna (*Thunnus albacares*) and Atlantic cod (*Gadus morhua*) (FAO, 2014). Cutlassfish is a popular food fish especially in Asia and usually cooked or served raw as sashimi (Nakamura & Parin, 1993).

Another fish species found in the same tropical upwelling region is the black snake mackerel *Nealotus tripes*, Johnson 1865 (Gempylidae). *N. tripes* can be found in mesopelagic habitats at depths to 600 m (Nakamura & Parin, 1993), with a global distribution in tropical and temperate waters (Strasburg, 1964; Yatsu et al., 2005; Tanaka, Mohri & Yamada, 2007; Mafalda Jr, Souza & Weiss, 2009; De Forest & Drazen, 2009; Wienerroither et al., 2009; Ivanov & Sukhanov, 2015). As a nyctoepipelagic species, it roams mesopelagic regions at daytime and migrates to the surface at night (e.g., Boehlert, Watson & Sun, 1992; Yatsu et al., 2005). Most gempylid species are not of commercial interest, except *Thyrsites atun* and *Rexea solandri* (Nakamura & Parin, 1993).

Marine ecosystems contain a broad diversity of parasites, which can serve as ecological indicators. Thus, parasites can be used as indicators for their host, reveal anthropogenic impact, e.g., accumulation of contaminants, or show systemic influences (Wood, 2007; Palm & Rückert, 2009; Palm, 2011; Wood et al., 2013; Wood et al., 2014). Moreover, the biodiversity of parasites in an ecosystem reflects the diversity of host organisms throughout its food web (Klimpel, Seehagen & Palm, 2003; Rückert, Palm & Klimpel, 2008). Combined with stomach content analyses of the hosts, parasitological data can therefore provide important insights into food webs, host-parasite relationship and host ecology in marine ecosystems. The parasite fauna and food ecology of *T. lepturus* have been studied before, however, there is a lack of data in the Canary Current System (CCS) (Timi, Martorelli & Sardella, 1999; Martins & Haimovici, 2000; Ho & Lin, 2002; Shih, 2004; Martins, Haimovici & Palacios, 2005; Bryan & Gill, 2007; Palm & Klimpel, 2007; Carvalho & Luque, 2011; Carvalho & Luque, 2012; Bueno, Aguiar & Santos, 2014). No data on the parasite fauna of *N. tripes* have been collected so far.

To gain insights into the CCS, the diet-composition and parasite fauna of *T. lepturus* and *N. tripes* from different habitats off Senegal and Mauritania were examined. The overall aim of this study was to assess whether the parasite fauna of pelagic fishes in the CCS represents the low species richness typically known from EBUEs (Angel, 1993; Sakko, 1998). Data from the commercially important *Trichiurus lepturus* were compared to findings from other studies in different coastal ecosystems.

## Material and Methods

**Figure 1:**
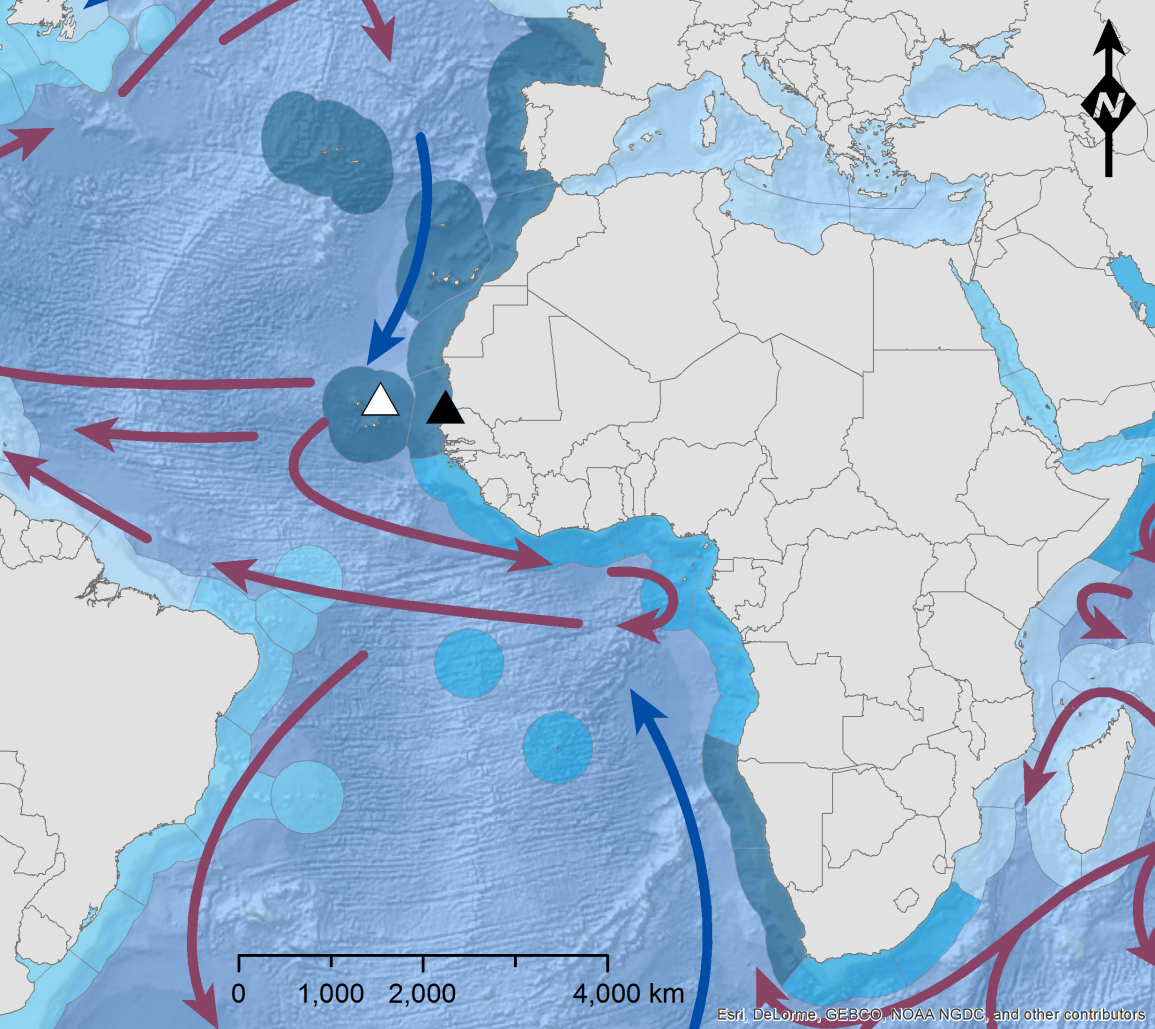
Map of the sampling area. Sampling area of *T. lepturus* (black triangle; haul from N16°45.49′, W16°38.16′to N16°43.34′, W16°39.76′) and *N. tripes* (white triangle; haul from N17°30.93′, W22°57.29′to N17°31.48′, W22°58.24′) off NW-Africa, with major ocean currents (blue arrow = cold; red arrow = warm; NOAA, National Weather Service, Maps.com) and coastal upwelling activity (increasing from light to dark blue; Hoekstra et al., 2010). The figure was built using ESRI ArcGIS 10.3 (ESRI, Redlands, CA, USA).

The samples of *T. lepturus* and *N. tripes* were taken during the 375th cruise of the research vessel Walther Herwig III. Sampling took place in the tropical East-Atlantic (FAO fishing area 34, 3.11) at different sampling sites off the North-West-African coast (Fig. 1). The catch was yielded by trawl fishing with a multisampler. After landing, the catch was sorted, identified to species level and weighed. The samples were stored in a freezer at −20 °C and defrosted over night at 4 °C in a refrigerator or 90 min at room temperature before examination. Specimens of *T. lepturus* (*n* = 104) and *N. tripes* (*n* = 91) were examined by assessing biometrical measures, stomach contents and parasite fauna of the fishes.

The stomach of each specimen was weighed full and empty to assess the total weight of its content. The different food components were sorted to the lowest possible taxonomic level, counted and weighed. Depending on the degree of digestion, prey numbers were identified by counting eyepairs (Crustacea), otoliths, vertebrae (Teleostei) and beaks (Cephalopoda). Trophic measures were calculated according to Hyslop (1980) for all specimens with stomachs containing food.

The body surface, fins, buccal cavity, nasal cavities and gills of the fishes were inspected for ectoparasites using a stereo microscope. Subsequently, the inner organs were examined for endoparasites. Parasites were stored in 99% ethanol for molecular identification or preserved in Roti^®^Histofix 4% (Carl Roth GmbH, Karlsruhe) for morphological examination and then cleared in glycerine and mounted on microscope slides. Parasitological measures were determined for each parasite taxon according to Bush et al. (1997).

Nematode larvae and myxozoans were identified by molecular methods. The DNA extraction from parasites was performed using a kit (PeqGOLD Microspin Tissue DNA Kit Protocol for small tissue sizes; Peqlab, VWR International GmbH, Darmstadt, Germany) and Acroprep plate (Pall Corporation, Port Washington, NY, USA). DNA samples were stored at 4 °C and processed promptly.

For the identification of the nematodes, the internal transcribed spacers and 5.8S rDNA were amplified, using primers TK1 (*Anisakis* spp.), NC5 (*Hysterothylacium* spp.) and NC2 (Zhu et al., 2000; Kuhn, García-Màrquez & Klimpel, 2011). A thermal cycler (Mastercycler, nexus gradient (eco); Eppendorf AG, Hamburg, Germany) was programmed to perform six steps: 1) heating (95 °C, 1 min), (2) denaturation (94 °C, 45 s), (3) annealing (55 °C, 45 s), (4) elongation (72 °C, 45 s), (5) (72 °C, 10 min) and (6) cooling (4 °C, ∞ min). Steps 2 to 4 were repeated in 40 cycles. PCR products were stored at 4 °C and processed promptly.

Myxozoan parasites were identified to species level using the 18S rDNA marker according to Shin et al. (2016) applying the primers Kudo-ShinF and Kudo-ShinR and the PCR-conditions described therein. A pairwise alignment was performed using the nucleotide Basic Local Alignment Search Tool (nBLAST, National Center for Biotechnology Information), searching NCBI Genbank for matches (Altschul et al., 1990).

A list of the parasites of *Trichiurus lepturus* was compiled by running a search on ‘Google-Scholar’ and ‘ISI-Web of Knowledge’ with the keyword combination “Trichiurus parasit*”. In addition, the Host-Parasite-Database of the National History Museum, London was checked for entries of *T. lepturus* and its synonym *T. haumela* as a host (Gibson, Bray & Harris, 2005). The taxon validity of the parasite records was checked using the World Register of Marine Species (WoRMS Editorial Board, 2017). There is no claim that the resulting list is comprehensive.

## Results

### Morphometrical data

Of the 104 examined *Trichiurus lepturus*, 59 individuals were male, 44 were female, and all gonads were premature. The mean total length (TL) of *T. lepturus* was 60.3 cm (±6.4 cm SD). The preanal length (PL) was approximately one third of the TL, with a median at 20.8 cm. The median total weight (TW) was 128.1 g and the median carcass weight (CW) was 120.3 g.

The sample of *N. tripes* (*n* = 91) was composed of 32 male and 58 female individuals. The median total length was 18.0 cm and the median preanal length was 11.1 cm. The median TW was 23.3 g and the median CW was 20.3 g. Detailed morphometric data are given in Table S1.

### Food ecology

Stomach content analyses revealed prey organisms from three taxonomic groups in both fish species, namely Mollusca, Crustacea and Teleostei (Tables 1 and 2).

**Table 1:** Diet composition of *Trichiurus lepturus*. Trophic measures of *T. lepturus* (*n* = 103) from the tropical East-Atlantic; number of stomachs (*n*), frequency of occurrence (F), weight percentage of prey (W), numerical percentage of prey (N) and index of relative importance (IRI).

Prey item	*n*	F [%]	W [%]	N [%]	IRI
**Mollusca**	8	7.7	2.67	0.020	20.99
Cephalopoda spp.	8	7.7	2.67	0.020	20.99
**Crustacea**	95	92.2	44.17	99.683	13,268.62
Decapoda spp.	2	0.9	0.04	0.002	0.05
Mysida spp.	95	92.2	44.12	99.660	13,261.90
**Teleostei**	54	52.4	53.20	0.310	2,805.41
Clupeiformes spp.	31	13.5	40.39	0.092	550.25
Trichiuridae sp.	2	1.9	0.2	0.005	0.54
indet.	38	52.4	12.5	0.211	470.29

**Table 2:** Diet composition of *N. tripes*. Trophic measures of *N. tripes* (*n* = 90) from the tropical East-Atlantic; number of stomachs (*n*), frequency of occurrence (F), weight percentage of prey (W), numerical percentage of prey (N) and index of relative importance (IRI).

Prey item	*n*	F [%]	W [%]	N [%]	IRI
**Mollusca**	2	2.2	0.5	1.66	4.83
Cephalopoda spp.	2	2.2	0.5	1.66	4.83
**Crustacea**	5	5.5	0.5	4.1	26.22
Decapoda spp.	4	3.3	0.5	3.33	17.27
Amphipoda sp.	1	1.1	0.001	0.83	0.92
**Teleostei**	90	100.0	98.9	94.17	19,310.32
Myctophidae spp.	15	16.6	31.6	14.16	764.05
indet.	75	83.3	67.9	84.95	15,293.88

The main diet organisms of *T. lepturus* were small, pelagic Crustacea. With a frequency of occurrence of 92.2% and a numerical percentage of prey of 99.6% Mysida were the most important food item of *T. lepturus* (IRI = 13,261.9). Fish prey occurred with a frequency of 52.4%, including 1.9% of possible cannibalism (Trichiuridae). Small bony fish had the highest weight percentage of prey (*W* = 53.2%), followed by the Mysida (*W* = 44.12%). Cephalopoda and Decapoda were rare food items in specimens examined in this study.

The main food items of *N. tripes* were small bony fish. In all examined stomachs containing food (90/91), small fish were identified. Fish was found to be the most important food item of the specimens examined in this study, by frequency (*F* = 100%), weight percentage (*W* = 98.9%) and numerical percentage (*N* = 94.17%). While a large proportion of fish prey was in an advanced stage of digestion, 17 prey fishes were identified as Myctophidae. Five examined stomachs contained Crustacea, a single amphipod and four decapods, and two stomachs contained Cephalopoda in advanced stages of digestion.

### Parasite fauna

Previous studies have identified more than 50 parasite taxa in six taxonomic groups parasitising *T. lepturus*. Most records are of digenetic trematodes, followed by nematodes (Table S2). In this study, the parasite fauna of *T. lepturus* consisted of at least nine different species belonging to six taxonomic groups (Table 3). The most frequent parasite was the digenean *Lecithochirium microstomum*, with a prevalence of 88.4% (*mI* = 58.6) in the stomach and rarely pyloric caeca. Ovigerous metacercarieae of a second digenean were less common and occurred with a prevalence of 7.6% (*mI* = 1.5) encapsulated in the body cavity and mesenteries. The intensity of digenean infection was positively correlated with the total length of the hosts (Spearman *r* = 0.72, *p* < 0.001). The parasite group with the highest intensity and abundance was the unidentified, early larval stages of cestodes. Unidentified tetraphyllidean cestode larvae occurred in the intestine and pylorus of 40.3% of the examined fish (*mI* = 19.6). Smaller fish were infested with higher intensities of cestode larvae than larger individuals (Spearman *r* =  − 0.39, *p* < 0.001). At least three nematode species of the families Anisakidae and Raphidascarididae were isolated from the body cavity and liver surface and identified molecularly. *Anisakis pegreffii* (*P* = 23%) and *A. typica* (*P* = 17.3%) occurred with similar prevalences and low intensities (*I* = 1–2 for both), while *Hysterothylacium* sp. was less frequent (*P* = 2.8%). Higher infection intensities (*I* = 1–4) were shown for very small, unidentified nematode larvae, that were encapsulated in the stomach tissue. The pylorus of one specimen of *T. lepturus* was infested with a single acanthocephalan.

**Table 3:** Parasite fauna of *T. lepturus*. Prevalence (P), intensity (I), mean intensity (mI), mean abundance (mA), site and stage of development.

Parasite taxon	site	stage	P [%]	*I*	*mI*	mA
**Digenea**						
*Lecithochirium microstomum*	s	A	88.4	1–222	58.6	51.85
indet.	bc, m	L	7.6	1–5	2.0	0.15
**Monogenea**						
*Octoplectanocotyla travassosi*	g	A	12.5	1–6	1.5	0.19
**Cestoda**						
Tetraphyllidea indet.	i	L	52.8	1–∼5,000	>900	>400
						
**Nematoda**						
*Anisakis pegreffii*	bc	L	23.0	1–2	2.0	0.26
*Anisakis typica*	bc	L	17.3	1–2	1.1	0.25
*Hysterothylacium* sp.	p, i	L	2.8	1	1.0	0.02
indet.	i, sc	L	25.0	1–14	2.0	0.5
**Acanthocephala**						
indet.	i		1.9	1	1.0	0.01
**Crustacea**						
Bomolochidae indet.	n	A	0.9	1	1	<0.01
Caligidae indet.	g	A	5.7	1–2	1.1	0.06

**Notes.**

Site abbreviations bc body cavity g gills gb gall bladder i intestine l liver m mesentery p pylorus s stomach sc stomach capsule
Stage of development A adult L larval

The only ectoparasites identified in this study were isolated from the gills. The monogenean *Octoplectanocotyla travassosi* occurred with a prevalence of 12.5% in *T. lepturus*, while Copepoda were present with a prevalence of 6.7%.

**Figure 2:**
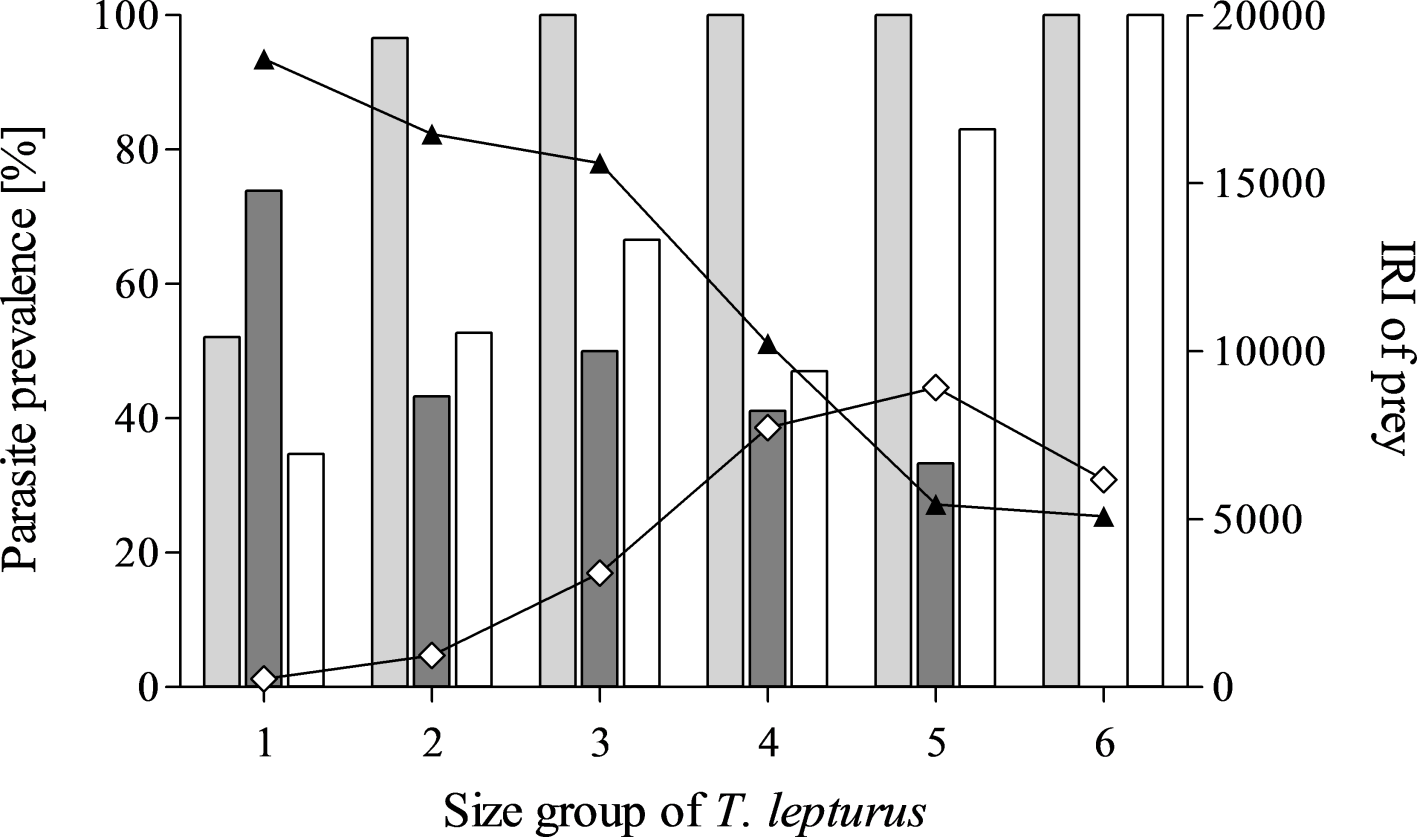
Parasite prevalence and prey importance in the size groups of *T. lepturus*. Prevalence of Digenea (light grey), Cestoda (dark grey) and Nematoda (white) and Index of Relative Importance (IRI) of Crustacea (black triangle) and Teleostei (white diamond) of different size groups of *T. lepturus*. Size group 1–6; <50 < 55 < 60 < 65 < 70 < 75 cm.

The diet composition of *T. lepturus* depended on its size. In larger specimens, the IRI of crustacean prey items decreased, while the IRI of teleost prey increased. The prevalence of the most important parasite taxa also varied between size groups of the fish host with increasing size, the prevalences of cestode larvae decreased, while digeneans and nematodes increased (Fig. 2).

The parasite fauna of *N. tripes* was composed of at least thirteen species from seven different taxonomic groups (Table 4). The muscle tissue of one specimen was infected with the myxozoan *Kudoa thyrsites*. The parasite was detected through a noticeable softening of the host muscle tissue. Digenean ovigerous metacercariae were the parasites with the highest prevalence in *N. tripes* (*P* = 62.9%) and occurred free or encysted with an intensity range between one and 82 parasites per host. Adult stages of this parasite were isolated from the body cavity, mesenteries and blood vessels. Digeneans from the family Hemiuridae were identified in two other fish specimens, encysted and degraded.

**Table 4:** Parasite fauna of *N. tripes*. Prevalence (P), intensity (I), mean intensity (mI), mean abundance (mA), site and stage of development.

Parasite taxon	Site	Stage	P [%]	I	mI	mA
**Myxozoa**						
*Kudoa thyrsites*	mu		1.0	–	–	–
**Digenea**						
indet.	bc, m, gb	L	69.2	1–82	9.6	6.64
Hemiuridae indet.		A	2.1	1	1	0.02
**Monogenea**						
Diclodophoridae indet.	g	A	58.2	1-7	1.5	0.92
**Cestoda**						
Tetraphyllidea indet.	p, gb, bd	L	58.2	1–235	55.6	32.3
indet.		L	42.8	1->9,000	907.1	220.40
*Nybelinia* sp.	sc	L	6.5	1–2	1.1	0.07
Trypanorhyncha spp.			3.2	1–2	1.3	0.04
**Nematoda**						
*Anisakis physeteris*		L	3.2	1–2	1.3	0.04
*Anisakis typica*	bc	L	15.3	1–2	1.0	0.16
*Anisakis* sp.		L	4.3	1–2	1.2	0.05
*Hysterothylacium* sp.	i	L	1.0	1	1.0	0.01
indet.	i, sc	L	20.8	1–35	3.8	0.81
**Acanthocephala**						
indet.	i		6.5	1	1.0	0.07
**Crustacea**						
Bomolochidae indet.	g	A	25.2	1–2	1.3	0.36
*Lernaeenicus* sp.	e	A	1.0	1	1.0	0.01

**Notes.**

Site abbreviations bc body cavity bd bile ducts e eye g gills gb gall bladder i intestine l liver m mesentery mu muscle p pylorus s stomach sc stomach cyst
Stage of development A adult L larval

Cestode larvae were the most abundant parasite group. Larvae isolated from the pylorus and intestine occurred with intensities up to 9,000 (estimate) (*P* = 24.1%). Unidentified tetraphyllidean cestode larvae were isolated from the pylorus, bile ducts and gall bladder. Cestodes from the family Trypanorhyncha were less common. Larval stages from the genus *Nybelinia* were isolated from cysts in the stomach wall (*P* = 6.5%). Nematodes from the families Anisakidae and Raphidascarididae were isolated from the body cavity of *N. tripes*. *A. typica* occurred with a prevalence of 15.3%. Furthermore, *A. physeteris*, *Anisakis* sp. and *Hysterothylacium* sp. were molecularly identified. Intensity of these nematode larvae ranged from one to two parasites per host. Minuscule larvae were isolated from cysts in the stomach wall (*P* = 20.8%).

Ectoparasites were isolated from the mouth and gills of *N. tripes*. Monogeneans from the family Diclidophoridae occurred with a prevalence of 58.2%. Parasitic crustaceans from the family Bomolochidae were less frequent (*P* = 25.2%).

## Discussion

Species richness in EBUEs is considered to be low in comparison to other tropical marine ecosystems (Angel, 1993; Sakko, 1998). Strong seasonal upwelling activities and circulation of cold, nutrient rich water do not only require adaption of the host organisms (e.g., fish spawning and recruitment), but also the parasite fauna (Carvalho & Luque, 2011; Cropper, Hanna & Bigg, 2014; Sambe et al., 2016; Tiedemann & Brehmer, 2017; Tiedemann et al., 2017). As a consequence, the parasite diversity found in EBUEs should be lower than in other coastal ecosystems. In the following paragraphs the parasite fauna as well as results of the stomach content analyses will be discussed separately for each of the two fish species from the CCS and, in the case of *Trichiurus lepturus,* compared with other studies from different coastal ecosystems (Table S2).

### Trichiurus lepturus

The parasite fauna of *T. lepturus* observed in this study was less diverse than in previous studies from other coastal regions in the Brazil Current and Kuroshio Current. Digeneans of the family Hemiuridae were the taxonomic group infesting *T. lepturus* with the highest prevalence. The diversity of digeneans described throughout the literature has a geographical pattern. There is a sampling bias towards studies from the Chinese Sea, probably due to the importance of *T. lepturus* as a food fish. In these studies, most species were described (Table S2). The species *L. microstomum* was the predominant parasite of *T. lepturus* in this study (*P* = 88.4%). A high mean intensity (*mI* = 58.61) reflects an aggregated occurrence of *L. microstomum*. Similar prevalences have been reported in *T. lepturus* sampled from coastal locations off Brazil (Carvalho & Luque, 2011; Bellay et al., 2011; Bueno, Aguiar & Santos, 2014). Bellay et al. (2011) characterised *L. microstomum* as a network hub species that is highly generalistic and parasitic in unrelated definitive host species. Such parasite species can be expected to thrive in EBUEs. *L. microstomum* has been described from several small fish species, e.g., *Engraulis anchoita*, which are typical prey organisms of adult *T. lepturus* (Timi, Martorelli & Sardella, 1999; Martins, Haimovici & Palacios, 2005). In this study, the prevalence and intensity of *L. microstomum* was shown to increase with the size of the host, while teleost prey gains importance (Fig. 2). Bueno, Aguiar & Santos (2014) described an ontogenetic shift in the diet of *T. lepturus*, from krill to fish, which is also supported by our data.

Cestode larvae occurred with high intensities of between one and >5,000 parasites per host individual. The relationship between the size of the fish and the intensity of cestode infection implies that smaller fish are more susceptible to higher infection intensities. This is likely influenced by either an ontogenetic change in its diet composition, or by the development of a host immune-response, or a combination of these factors (e.g., Stunkard, 1977; Marcogliese, 1995; Buchmann, 2012). This hypothesis is also supported by the findings of previous studies examining larger specimens, where reported infection intensities were much lower and the cestode larvae were further developed (Shih, 2004; Carvalho & Luque, 2011; Bueno, Aguiar & Santos, 2014). It is also possible that the density of cestode larvae varies according to the upwelling seasonality (Carvalho & Luque, 2011). In order to examine this hypothesis, samples need to be collected during and between recurring upwelling events. Taking previous research into account, a higher diversity of cestode larvae would have been expected in *T. lepturus* (Table S2) (Shih, 2004; Carvalho & Luque, 2011; Bueno, Aguiar & Santos, 2014).

*Trichiurus lepturus* has been described as host organism of various nematodes (Table S2) and serves as an intermediate host for nematodes identified in the present study. As the data in this study show, *T. lepturus* could accumulate anisakid nematode larvae with increasing size and age, when fishes gain importance as prey, and then serve as a paratenic host (Martins, Haimovici & Palacios, 2005). *Anisakis* spp. have a pelagic life cycle with Crustacea as first intermediate hosts, matching the epipelagic feeding behaviour of *T. lepturus*, which feeds on large batches of euphausiids and small fish (Nakamura & Parin, 1993; Abollo, Gestal & Pascual, 2001; Mattiucci & Nascetti, 2006). *A. pegreffii* is closely related to *A. simplex* (*s.s.*) and commonly found in mid-Atlantic waters and the Mediterranean (Klimpel et al., 2010; Mattiucci et al., 2013). Fish specimens in the present study were caught considerably further south than the southern limit of *A. simplex* (*s.s.*) (Kuhn et al., 2013). Implied by the absence of *A. simplex* (*s.s.*) in the two fish hosts, the fish from the present sample did not migrate further north than Gibraltar. Kong et al. (2015) identified *A. pegreffii* with a prevalence of 84.4% in adult *T. lepturus* from Chinese waters and *Anisakis simplex* (*s.s.*) and *A. typica* were detected with lower prevalences of 0.6% and 1.5% respectively. In comparison, the prevalence of *A. typica* was high in the present study Borges et al. (2012) found higher prevalences of *A. typica* (20.3%) and *Hysterothylacium* sp. (51.3%) in the Brazil Current region. Another study from this region recorded prevalences of anisakid nematode larvae in *T. lepturus* ranging from 73.3 to 100% throughout the seasons (Carvalho & Luque, 2011). The different prevalences of *Anisakis* spp. can be explained by a location effect and the availability of the definitive hosts (toothed whales, mainly delphinids), e.g., coastal dwelling dolphins for *A. typica* (Mattiucci & Nascetti, 2008). As *Hysterothylacium* is transmitted to fish through (crustacean) invertebrate hosts, the higher prevalence found by Borges et al. (2012) may be connected to the feeding habits of the fish. Also, Køie (1993) showed that the size of the larvae of *H. aduncum* during ingestion of the crustacean host by a fish might be crucial to the parasite’s survival.

The nematode fauna of *T. lepturus* has a zoonotic potential because of the occurrence of *A. pegreffii*, which may cause anisakiasis. An infection may go along with gastro-intestinal or allergic symptoms (Zhu et al., 1998; Daschner & Pascual, 2005; Nieuwenhuizen et al., 2006; Hochberg & Hamer, 2010; Mattiucci et al., 2013). *A. pegreffii* is the main cause of anisakiasis in the Mediterranean (Mattiucci et al., 2013). If located on the visceral surface of the intestine, migration of the parasite into the muscle tissue of the fillet might be favoured, resulting in a possible health hazard (Klapper et al., 2015; Mladineo et al., 2017). However, the risk of ingesting a living larva from a correctly gutted fish can be considered as rather small. This is due to the body shape of *T. lepturus* and the position of its body cavity which only expands over a third of the total length of the fish. Nematode larvae can migrate into the muscle but are mostly found in the belly flaps of the fish, which are usually not prepared as a food (Klapper et al., 2015). To assess whether nematodes migrate into the muscle tissue of *T. lepturus*, appropriate techniques e.g., pepsin-HCl-digestion should be applied. The presence of the larvae in the visceral tissue of *T. lepturus* can be conceived as potentially hazardous with respect to food allergies in sensitized individuals (Nieuwenhuizen et al., 2006; Mattiucci et al., 2013).

The infestation with ectoparasites is most likely due to the schooling behaviour of subadult *T. lepturus*, which provides good conditions for the transmission of monoxenic monogeneans and Copepoda (Hunter, 1966; Carvalho & Luque, 2011; Johnson et al., 2011). In comparison to other studies, ectoparasites were scarce (Carvalho & Luque, 2011; Carvalho & Luque, 2012; Bueno, Aguiar & Santos, 2014). *Trichiurus lepturus* sampled from the warm Brazil Current, which has a rather moderate upwelling activity, had a higher diversity of ectoparasites (three monogenean species, two copepod species) than the specimens from the Canary Current examined in this study (one monogenean species, one copepod species) (Bueno, Aguiar & Santos, 2014). From this finding we conclude that the intense seasonal upwelling activity in the CCS-EBUE might diminish parasite diversity and promote generalist species.

### N. tripes

The newly recorded parasite fauna of *N. tripes* was dominated by three taxa, digenean larvae (mostly encysted ovigerous metacercariae), tetraphyllidean cestode larvae and a monogenean species from the family Diclidophoridae. The presence of digenean larvae suggests benthic feeding behaviour, because gastropods are obligatory intermediate hosts. As a pelagic species with a diet composed of a large proportion of teleost prey, their route of infection for *N. tripes* is most likely through a teleost transport host. Previous studies have shown, that preadult stages of the genus *Lecithochirium* occur encysted in the viscera of the host (Gibson & Bray, 1986). Free stages in the intestine are possibly transitioning to the viscera, where they might encapsulate (Gibson & Bray, 1986). Thus, the digenean trematodes extracted from our specimens could belong to this genus.

The cestodes isolated from *N. tripes* provide a similar picture to that in *T. lepturus*. High intensities of small unidentifiable larvae were isolated from the digestive system. The larval tetraphyllidean cestode morphotaxon *Scolex pleuronectis* was previously recorded, but no peer-reviewed parasitological studies on *N. tripes* have been published. The bile ducts and gall bladder as sites of infection for *tetraphyllidean cestode larvae* have been observed in other fish species (e.g., *Hippoglossus stenolepis*, Blaylock, Holmes & Margolis, 1998) and are identified in *N. tripes* for the first time. Many aspects of the life-cycle of tetraphyllidean cestodes are still unknown, but most species have elasmobranch definitive hosts (Marcogliese, 1995). This indicates that *T. lepturus* and *N. tripes* may be important prey organisms for pelagic sharks in this area. In contrast to *T. lepturus*, no connection between the cestode infection intensity and the length and weight of the fish could be made. A possible reason for the high abundance of cestode larvae is the piscivorous diet. A parasite fauna of larval cestodes and nematodes and low species richness has been described as the typical parasite fauna of myctophid fishes, the main diet component of *N. tripes* (Klimpel, Kellermanns & Palm, 2008). Myctophids play an important role in the transmission of parasites throughout the water column, especially nematodes from the genus *Anisakis* (Klimpel, Kellermanns & Palm, 2008). Mateu et al. (2015) described Myctophidae as a link in the life-cycle of *A. physeteris*, connecting the crustacean host to the squid, which are consumed by the definitive host, the sperm whale *Physeter macrocephalus* (Mattiucci & Nascetti, 2008; Mateu et al., 2015 and the references therein). It seems that *N. tripes* plays a similar role as Myctophidae, as they also occupy an intermediate position in the food web (linking zooplankton to large vertebrates) and perform diurnal vertical migrations (Boehlert, Watson & Sun, 1992; Yatsu et al., 2005). The presence of *A. physeteris* and *A. typica* in our sample is an indicator of this, as the former is host specific to *P. macrocephalus*, which inhabits deeper water layers and the latter is host specific to dolphins that prefer the epipelagic (Mattiucci & Nascetti, 2008; Mateu et al., 2015). It also indicates that *N. tripes* might be preyed upon by squid.

The monogenean infestation of *N. tripes* is an indicator of schooling-behaviour. Many monogenean species are host specific, their adhesive organs (haptoral clamps) being adapted to the gills of the host (Boeger & Kritsky, 1993). Thus, it is possible that the present diclidophorid parasite species has not yet been described. *N. tripes* is a new host record for *K. thyrsites*, *A. typica* and *A. physeteris*.

### Parasite richness

**Figure 3:**
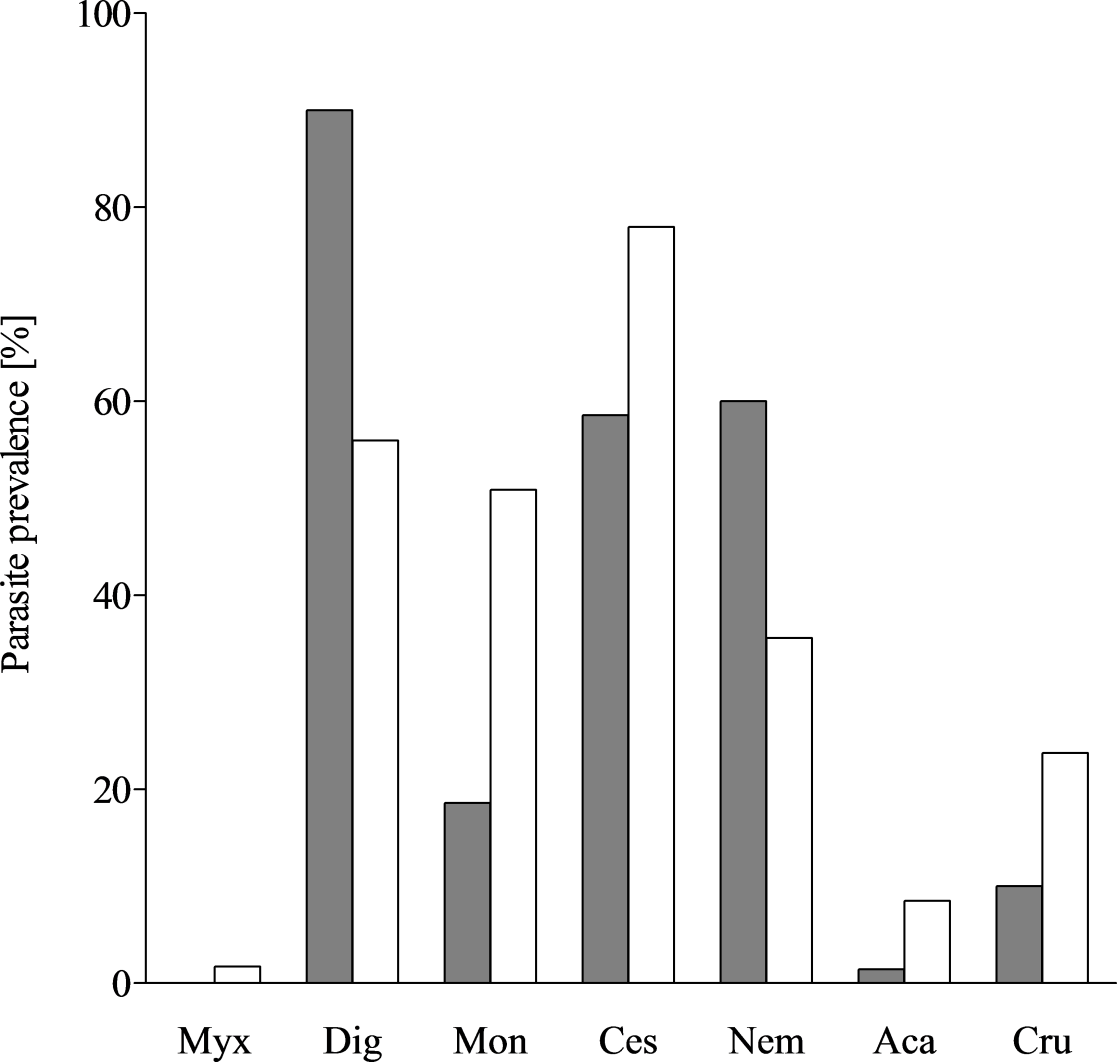
Parasite prevalence in *T. lepturus* and *N. tripes*. Prevalence of parasite taxa from *T. lepturus* (grey) and *N. tripes* (white). Myx, Myxosporea; Dig, Digenea; Mon, Monogenea; Ces, Cestoda; Nem, Nematoda; Aca, Acanthocephala; Cru, Crustacea.

Due to the distribution of biodiversity throughout the water column, the parasite diversity was expected to be lower in the mesopelagial (*N. tripes*) than in the epipelagial (*Trichiurus lepturus*) (Angel, 1993). Both examined fish species have a distinct parasite fauna, the mesopelagic species *N. tripes* being infested by 13 parasite taxa from seven different groups, whereas *T. lepturus* was infested by at least nine parasite taxa from 6 different groups (Fig. 3). This finding contradicted the expectation, but as *N. tripes* is nyctoepipelagic, both species likely feed in the epipelagial. The differences in their parasitisation could be explained through the results of the stomach content analyses. With a piscivorous diet, *N. tripes* consumed mostly prey from a higher trophic level than *T. lepturus*, which had a large proportion of zooplankton in its diet. Thus, *N. tripes* is more likely to accumulate different parasites through its diet. The parasites occurring in both species were digenean larvae, early stages of cestode larvae, *Anisakis typica* and *Hysterothylacium* sp. The discovery of these parasites in both fishes examined is in accordance with the generalist lifestyle of the parasites and their records from various teleost hosts (e.g., Klimpel & Rückert, 2005; Carreras-Aubets et al., 2012; Kuhn et al., 2013). *Anisakis typica* is the dominant *Anisakis* species in tropical shelf regions (Mattiucci et al., 2002; Kuhn et al., 2013). The definitive hosts of *A. typica* are typically small coastal dwelling cetaceans which belong to the families Delphinidae, Phocoenidae and Pontoporiidae (Mattiucci et al., 2002). The fauna of small coastal Cetacea of the sampling area offers a diverse range of possible definitive hosts for *A. typica*, which is reflected in its relatively high prevalence in both *T. lepturus* and *N. tripes* (Dupuy & Maigret, 1976; Smeenk, Leopold & Addink, 1992; Hutterer, 1994; Robineau & Vely, 1997; Van Waerebeek et al., 1999; Felix & Van Waerebeek, 2005; Wenzel et al., 2009; Weir et al., 2011; Weir et al., 2014; Jung et al., 2016). A predator–prey relationship between dolphins from the genus *Sotalia* spp., which are a definitive host for *A. typica*, and *T. lepturus* was previously described (Melo et al., 2006; Carvalho et al., 2008). As a typical shelf inhabitant with a broad spectrum of intermediate hosts and pelagic life-cycle, infestation with *A. typica* was expected in the studied fishes.

## Conclusion

The discovery of mainly generalist parasites along with larval anisakid nematodes and cestode larvae reflects the typical parasite infestation of mesopredatory pelagic fishes. The abundance of nematode larvae is connected to the lower trophic level of the fishes, which consumed mainly planktonic Crustacea (*T. lepturus*) and small Teleostei (*N. tripes*). The low diversity and the presence of generalist parasites represents the species composition of an eastern boundary upwelling ecosystem: few dominant generalists with a high abundance, *L. microstomum*, digenean larvae and larval cestodes and a low overall species richness. An effect of upwelling seasonality on the parasite fauna could be examined in future studies.

##  Supplemental Information

10.7717/peerj.5339/supp-1 Table S1 Morphometric measures of *Trichiurus lepturus* and *Nealotus tripes* ci, confidence interval; CW, carcass weight; GW, gonad weight; LW, liver weight; max, maximum; min, minimum; *N.l.*, *N. tripes*; SD, standard deviation; SL, standard length; TL, total length; *T.l.*, *T. lepturus*; TW, total weight.

10.7717/peerj.5339/supp-2 Table S2 Parasite fauna of *Trichiurus lepturus* according to online research Reported parasites of *T. lepturus*, parasite taxa and prevalences (if available) from the literature with corresponding references. Asterisks indicate parasites discovered in the present study. BC, Brazil Current; CC, Canary Current; FC, Falkland Current; KC, Kuroshio Current; P, Prevalence; TWC, Taiwan Warm Current; ZMCC, Zhe-Min Coastal Current.

10.7717/peerj.5339/supp-3 Data S1 Raw data of morphometrics and diet of *Trichiurus lepturus* and *Nealotus tripes* specimens ID, host identification code, Hol., catch number; SL, standard lenght; TL, total length; PL, preanal length; TW, total weight; CW, carcass weight; GW, gonad weight; LW, liver weight; SW, stomach weight.

10.7717/peerj.5339/supp-4 Data S2 Raw data of the parasitisation of *Trichiurus lepturus* and *Nealotus tripes* specimens Ac, Acanthocephala, *A. peg*, *Anisakis pegreffii*, *A. phy*, *Anisakis physeteris*, *A*. sp., *Anisakis* sp., *A. typ*, *Anisakis typica*; Bo, Bomolochidae; C, Cestoda; Cal , Caligidae; Cr, Crustacea; D, Digenea; Dic, Diclidophoridae, Hem, Hemiuridae, *H*. sp., Hysterothylacium sp.; ID, host identification code; *K. thy*, *Kudoa thyrsites*; *L. mic*, *Lecithochirium microstomum*; *L*. sp., *Lernaeenicus* sp.; M, Monogenea; My, Myxozoa; N, Nematoda; *N*. sp., *Nybelinia* sp.; *O. tra*, *Octoplectanocotyle travassosi*; Tet , Tetraphyllidea; Try, Trypanorhyncha.

10.7717/peerj.5339/supp-5 Data S3 DNA sequences used for molecular parasite identification in .fasta format (not aligned)

10.7717/peerj.5339/supp-6 Data S4 Parasite specimens from *Trichiurus lepturus* deposited in the scientific collection of the Senckenberg Research Institute, Frankfurt am Main, Germany

## Additional Information and Declarations

### Data Availability

The following information was supplied regarding data availability: The raw data are provided in the Supplemental Files.

## References

[ref-1] Abollo E, Gestal C, Pascual S (2001). *Anisakis* infestation in marine fish and cephalopods from Galician waters: an updated perspective. Parasitology Research.

[ref-2] Altschul SF, Gish W, Miller W, Myers EW, Lipman DJ (1990). Basic local alignment search tool. Journal of Molecular Biology.

[ref-3] Angel MV (1993). Biodiversity of the Pelagic Ocean. Conservation Biology.

[ref-4] Bellay S, Lima DP, Takemoto RM, Luque JL (2011). A host-endoparasite network of Neotropical marine fish: are there organizational patterns?. Parasitology.

[ref-5] Blaylock RB, Holmes JC, Margolis L (1998). The parasites of Pacific halibut (*Hippoglossus stenolepis*) in the eastern North Pacific: host-level influences. Canadian Journal of Zoology.

[ref-6] Boeger W, Kritsky D (1993). Phylogeny and a revised classification of the Monogenoidea Bychowsky, 1937 (platyhelminthes). Systematic Parasitology.

[ref-7] Boehlert GW, Watson W, Sun LC (1992). Horizontal and vertical distributions of larval fishes around an isolated oceanic island in the tropical Pacific. Deep Sea Research Part A. Oceanographic Research Papers.

[ref-8] Borges JN, Cunha LFG, Santos HLC, Monteiro-Neto C, Santos CP (2012). Morphological and molecular diagnosis of Anisakid Nematode larvae from Cutlassfish (*Trichiurus lepturus*) off the Coast of Rio de Janeiro, Brazil. PLOS ONE.

[ref-9] Bryan DR, Gill SM (2007). Seasonal occurrence of Atlantic cutlassfish, *Trichiurus lepturus*, in southeastern Florida with notes on reproduction and stomach contents. Florida Scientist.

[ref-10] Buchmann K (2012). Fish immune responses against endoparasitic nematodes—experimental models. Journal of Fish Diseases.

[ref-11] Bueno GBF, Aguiar JCC, Santos SMC dos (2014). Community structure of metazoan parasites of *Trichiurus lepturus* (Perciformes, Trichiuridae) from Ubatuba, Southwestern Atlantic Ocean, Brazil. Acta Scientiarum. Biological Sciences.

[ref-12] Bush AO, Lafferty KD, Lotz JM, Shostak AW (1997). Parasitology meets ecology on its own terms: Margolis et al. revisited. The Journal of Parasitology.

[ref-13] Carreras-Aubets M, Montero FE, Kostadinova A, Gibson DI, Carrassón M (2012). Redescriptions of two frequently recorded but poorly known hemiurid digeneans, *Lecithochirium musculus* (Looss, 1907) (Lecithochiriinae) and *Ectenurus lepidus Looss*, 1907 (Dinurinae), based on material from the western Mediterranean. Systematic Parasitology.

[ref-14] Carvalho CEV, Di Beneditto APM, Souza CMM, Ramos RMA, Rezende CE (2008). Heavy metal distribution in two cetacean species from Rio de Janeiro State, south-eastern Brazil. Journal of the Marine Biological Association of the United Kingdom.

[ref-15] Carvalho AR, Luque JL (2011). Seasonal variation in metazoan parasites of *Trichiurus lepturus* (Perciformes: Trichiuridae) of Rio de Janeiro, Brazil. Brazilian Journal of Biology.

[ref-16] Carvalho AR, Luque JL (2012). Three new species of monogeneans parasitic on Atlantic cutlassfish *Trichiurus lepturus* (Perciformes: Trichiuridae) from Southeastern Brazil. Acta Scientiarum. Biological Sciences.

[ref-17] Cropper TE, Hanna E, Bigg GR (2014). Spatial and temporal seasonal trends in coastal upwelling off Northwest Africa, 1981–2012. Deep Sea Research Part I: Oceanographic Research Papers.

[ref-18] Daschner A, Pascual CY (2005). *Anisakis simplex*: sensitization and clinical allergy. Current Opinion in Allergy and Clinical Immunology.

[ref-19] De Forest L, Drazen J (2009). The influence of a Hawaiian seamount on mesopelagic micronekton. Deep Sea Research Part I: Oceanographic Research Papers.

[ref-20] Dupuy AR, Maigret J (1976). The marine mammals of the senegal coasts part 1 schedule of observations between 1960 and 1976. Bulletin de l’Institut Fondamental d’Afrique Noire Serie A Sciences Naturelles.

[ref-21] Faye S, Lazar A, Sow BA, Gaye AT (2015). A model study of the seasonality of sea surface temperature and circulation in the Atlantic North-eastern tropical upwelling system. Atmospheric Science.

[ref-22] Felix F, Van Waerebeek K (2005). Whale mortality from ship strikes in Ecuador and West Africa. Latin American Journal of Aquatic Mammals.

[ref-23] Food and Agriculture Organization of the United Nations (FAO) (2009). Report of the FAO working group on the assessment of small Pelagic Fish off Northwest Africa. Nouakchott, Mauritania, 21–30 April 2009. http://www.fao.org/docrep/014/i2237b/i2237b00.htm.

[ref-24] Food and Agriculture Organization of the United Nations (FAO) (2012). FAO Fisheries and Aquaculture Department. FAO yearbook. Fishery and aquaculture statistics. http://www.fao.org/3/a-i3740t.pdf.

[ref-25] Food and Agriculture Organization of the United Nations (FAO) (2014). FAO Fisheries and Aquaculture Department. Summary tables of Fishery Statistics. http://www.fao.org/fishery/docs/STAT/summary/default.htm.

[ref-26] Gibson DI, Bray RA (1986). The Hemiuridae (Digenea) of fishes from the north-east Atlantic. Bulletin of the British Museum.

[ref-27] Gibson DI, Bray RA, Harris EA (2005). Host-parasite database of the Natural History Museum, London. http://www.nhm.ac.uk/research-curation/scientific-resources/taxonomy-systematics/host-parasites/.

[ref-28] Ho J, Lin CL (2002). New species of *Metacaligus* (Caligidae, Copepoda) parasitic on the Cutlassfish (*Trichiurus lepturus*) of Taiwan, with a cladistic analysis of the family Caligidae. Zoological Science.

[ref-29] Hochberg NS, Hamer DH (2010). Anisakidosis: perils of the deep. Clinical Infectious Diseases.

[ref-30] Hoekstra JM, Molnar JL, Jennings M, Revenga C, Spalding MD, Boucher TM, Robertson JC, Heibel TJ, Ellison K (2010). The atlas of global conservation: changes, challenges, and opportunities to make a difference.

[ref-31] Hunter JR (1966). Procedure for analysis of schooling behavior. Journal of the Fisheries Research Board of Canada.

[ref-32] Hutterer R (1994). Dwarf sperm whale *Kogia simus* in the Canary Islands. Lutra.

[ref-33] Hyslop EJ (1980). Stomach contents analysis—a review of methods and their application. Journal of Fish Biology.

[ref-34] Ivanov OA, Sukhanov VV (2015). Species structure of pelagic ichthyocenes in Russian waters of Far Eastern seas and the Pacific Ocean in 1980–2009. Journal of Ichthyology.

[ref-35] Johnson MB, Lafferty KD, Van Oosterhout C, Cable J (2011). Parasite transmission in social interacting hosts: monogenean epidemics in Guppies. PLOS ONE.

[ref-36] Jung JL, Mullie WC, Van Waerebeek K, Wagne MM, Bilal ASO, Sidaty ZEAO, Toomey L, Meheust E, Marret F (2016). Omura’s whale off West Africa: autochthonous population or inter-oceanic vagrant in the Atlantic Ocean?. Marine Biology Research.

[ref-37] Klapper R, Kuhn T, Münster J, Levsen A, Karl H, Klimpel S (2015). Anisakid nematodes in beaked redfish (*Sebastes mentella*) from three fishing grounds in the North Atlantic, with special notes on distribution in the fish musculature. Veterinary Parasitology.

[ref-38] Klimpel S, Busch MW, Sutton T, Palm HW (2010). Meso- and bathy-pelagic fish parasites at the Mid-Atlantic Ridge (MAR): low host specificity and restricted parasite diversity. Deep Sea Research Part I: Oceanographic Research Papers.

[ref-39] Klimpel S, Kellermanns E, Palm HW (2008). The role of pelagic swarm fish (Myctophidae: Teleostei) in the oceanic life cycle of Anisakis sibling species at the Mid-Atlantic Ridge, Central Atlantic. Parasitology Research.

[ref-40] Klimpel S, Rückert S (2005). Life cycle strategy of *Hysterothylacium aduncum* to become the most abundant anisakid fish nematode in the North Sea. Parasitology Research.

[ref-41] Klimpel S, Seehagen A, Palm HW (2003). Metazoan parasites and feeding behaviour of four small-sized fish species from the central North Sea. Parasitology Research.

[ref-42] Køie M (1993). Aspects of the life cycle and morphology of *Hysterothylacium aduncum* (Rudolphi, 1802) (Nematoda, Ascaridoidea, Anisakidae). Canadian Journal of Zoology.

[ref-43] Kong Q, Fan L, Zhang J, Akao N, Dong K, Lou D, Ding J, Tong Q, Zheng B, Chen R, Ohta N, Lu S (2015). Molecular identification of Anisakis and *Hysterothylacium* larvae in marine fishes from the East China Sea and the Pacific coast of central Japan. International Journal of Food Microbiology.

[ref-44] Kuhn T, García-Màrquez J, Klimpel S (2011). Adaptive radiation within Marine Anisakid Nematodes: a zoogeographical modeling of cosmopolitan, Zoonotic parasites. PLOS ONE.

[ref-45] Kuhn T, Hailer F, Palm HW, Klimpel S (2013). Global assessment of molecularly identified *Anisakis* Dujardin, 1845 (Nematoda: Anisakidae) in their teleost intermediate hosts. Folia Parasitologica.

[ref-46] Mafalda Jr P, Souza CMM, Weiss G (2009). Composition of Trichiuridae and Gempylidae larvae (Teleostei) and their association with water masses in the Southwest Atlantic Ocean. Oceanological and Hydrobiological Studies.

[ref-47] Marcogliese DJ (1995). The role of zooplankton in the transmission of helminth parasites to fish. Reviews in Fish Biology and Fisheries.

[ref-48] Martins AS, Haimovici M (2000). Reproduction of the cutlassfish *Trichiurus lepturus* in the southern Brazil subtropical convergence ecosystem. Scientia Marina.

[ref-49] Martins AS, Haimovici M, Palacios R (2005). Diet and feeding of the cutlassfish *Trichiurus lepturus* in the subtropical convergence ecosystem of southern Brazil. Journal of the Marine Biological Association of the United Kingdom.

[ref-50] Mateu P, Nardi V, Fraija-Fernández N, Mattiucci S, Gil de Sola L, Raga JA, Fernández M, Aznar FJ (2015). The role of lantern fish (Myctophidae) in the life-cycle of cetacean parasites from western Mediterranean waters. Deep Sea Research Part I: Oceanographic Research Papers.

[ref-51] Mattiucci S, Fazii P, De Rosa A, Paoletti M, Megna AS, Glielmo A, De Angelis M, Costa A, Meucci C, Calvaruso V, Sorrentini I, Palma G, Bruschi F, Nascetti G (2013). Anisakiasis and gastroallergic reactions associated with *Anisakis pegreffii* infection, Italy. Emerging Infectious Diseases.

[ref-52] Mattiucci S, Nascetti G (2006). Molecular systematics, phylogeny and ecology of anisakid nematodes of the genus *Anisakis* Dujardin, 1845: an update. Parasite.

[ref-53] Mattiucci S, Nascetti G (2008). Advances and trends in the molecular systematics of anisakid nematodes, with implications for their evolutionary ecology and host-parasite co-evolutionary processes. Advances in Parasitology.

[ref-54] Mattiucci S, Paggi L, Nascetti G, Santos CP, Costa G, Beneditto APD, Ramos R, Argyrou M, Cianchi R, Bullini L (2002). Genetic markers in the study of *Anisakis typica* (Diesing, 1860): larval identification and genetic relationships with other species of *Anisakis* Dujardin, 1845 (Nematoda: Anisakidae). Systematic Parasitology.

[ref-55] Mbaye BC, Brochier T, Echevin V, Lazar A, Lévy M, Mason E, Gaye AT, Machu E (2015). Do *Sardinella aurita* spawning seasons match local retention patterns in the Senegalese-Mauritanian upwelling region?. Fisheries Oceanography.

[ref-56] Meissa B, Gascuel D, Rivot E (2013). Assessing stocks in data-poor African fisheries: a case study on the white grouper *Epinephelus aeneus* of Mauritania. African Journal of Marine Science.

[ref-57] Melo OP, Ramos RMA, Beneditto D, Madeira AP (2006). Helminths of the marine tucuxi, *Sotalia fluviatilis* (Gervais, 1853)(Cetacea: Delphinidae), in northern Rio de Janeiro State, Brazil. Brazilian Archives of Biology and Technology.

[ref-58] Mladineo I, Bušelić I, Hrabar J, Vrbatović A, Radonić I (2017). Population parameters and mito-nuclear mosaicism of *Anisakis* spp. in the Adriatic Sea. Molecular and Biochemical Parasitology.

[ref-59] Nakamura I, Parin NV (1993). Snake mackerels and cutlassfishes of the world (families Gempylidae and Trichiuridae): an annotated and illustrated catalogue of the snake mackerels, snoeks, escolars, gemfishes, sackfishes, domine, oilfish, cutlassfishes, scabbardfishes, hairtails, and frostfishes known to date.

[ref-60] Nieuwenhuizen N, Lopata AL, Jeebhay MF, Herbert DR, Robins TG, Brombacher F (2006). Exposure to the fish parasite *Anisakis* causes allergic airway hyperreactivity and dermatitis. Journal of Allergy and Clinical Immunology.

[ref-61] Palm HW, Mehlhorn H (2011). Fish parasites as biological indicators in a changing world: can we monitor environmental impact and climate change?. Progress in parasitology.

[ref-62] Palm HW, Klimpel S (2007). Evolution of parasitic life in the ocean. Trends in Parasitology.

[ref-63] Palm HW, Rückert S (2009). A new approach to visualize ecosystem health by using parasites. Parasitology Research.

[ref-64] Robineau D, Vely M (1997). Preliminary data (body size, craniometry) on Tursiops truncatus from north western African coasts (Mauritania, Senegal). Mammalia.

[ref-65] Rückert S, Palm HW, Klimpel S (2008). Parasite fauna of seabass (*Lates calcarifer*) under mariculture conditions in Lampung Bay, Indonesia. Journal of Applied Ichthyology.

[ref-66] Sakko AL (1998). The influence of the Benguela upwelling system on Namibia’s marine biodiversity. Biodiversity & Conservation.

[ref-67] Sambe B, Tandstad M, Caramelo AM, Brown BE (2016). Variations in productivity of the Canary Current Large Marine Ecosystem and their effects on small pelagic fish stocks. Environmental Development.

[ref-68] Shih HH (2004). Parasitic helminth fauna of the cutlass fish, *Trichiurus lepturus* L., and the differentiation of four anisakid nematode third-stage larvae by nuclear ribosomal DNA sequences. Parasitology Research.

[ref-69] Shin SP, Shirakashi S, Hamano S, Kato K, Lasso LT, Yokoyama H (2016). Phylogenetic study of the genus *Kudoa* (Myxozoa: Multivalvulida) with a description of Kudoa rayformis sp. nov. from the trunk muscle of Pacific sierra *Scomberomorus sierra*. Molecular Phylogenetics and Evolution.

[ref-70] Smeenk C, Leopold MF, Addink MJ (1992). Note on the harbour porpoise *Phocoena phocoena* in Mauritania, West Africa. Lutra.

[ref-71] Strasburg DW (1964). Postlarval Scombroid fishes of the genera *Acanthocybium*, *Nealotus*, and *Diplospinus* from the Central Pacific Ocean. Pacific Science.

[ref-72] Stunkard HW (1977). Studies on Tetraphyllidean and Tetrarhynchidean Metacestodes from Squids taken on the New England Coast. Biological Bulletin.

[ref-73] Tanaka Y, Mohri M, Yamada H (2007). Distribution, growth and hatch date of juvenile Pacific bluefin tuna *Thunnus orientalis* in the coastal area of the sea of Japan. Fisheries Science.

[ref-74] Tiedemann M, Brehmer P (2017). Larval fish assemblages across an upwelling front: indication for active and passive retention. Estuarine, Coastal and Shelf Science.

[ref-75] Tiedemann M, Fock HO, Brehmer P, Döring J, Möllmann C (2017). Does upwelling intensity determine larval fish habitats in upwelling ecosystems? The case of Senegal and Mauritania. Fisheries Oceanography.

[ref-76] Timi JT, Martorelli SR, Sardella NH (1999). Digenetic trematodes parasitic on *Engraulis anchoita* (Pisces: Engraulidae) from Argentina and Uruguay. Folia Parasitologica.

[ref-77] Van Camp L, Nykjaer L, Mittelstaedt E, Schlittenhardt P (1991). Upwelling and boundary circulation off Northwest Africa as depicted by infrared and visible satellite observations. Progress in Oceanography.

[ref-78] Van Waerebeek K, Andre M, Sequeira M, Martin V, Robineau D, Collet A, Papastavrou V, Ndiaye E (1999). Spatial and temporal distribution of the minke whale, *Balaenoptera acutorostrata* (Lacepede, 1804), in the southern northeast Atlantic Ocean and the Mediterranean Sea, with reference to stock identity. Journal of Cetacean Research and Management.

[ref-79] Weir CR, Coles P, Ferguson A, May D, Baines M, Figueirdo I, Reichelt M, Goncalves L, Boer MN de, Rose B, Edwards M, Travers S, Ambler M, Félix H, Wall D, Azhakesan VAA, Betenbaugh M, Fennelly L, Haaland S, Hak G, Juul T, Leslie RW, McNamara B, Russell N, Smith JA, Tabisola HM, Teixeira A, Vermeulen E, Vines J, Williams A (2014). Clymene dolphins (*Stenella clymene*) in the eastern tropical Atlantic: distribution, group size, and pigmentation pattern. Journal of Mammalogy.

[ref-80] Weir CR, Van Waerebeek K, Jefferson TA, Collins T (2011). West Africa’s Atlantic humpback dolphin (*Sousa teuszii*): endemic, enigmatic and soon endangered?. African Zoology.

[ref-81] Wenzel FW, Allen J, Berrow S, Hazevoet CJ, Jann B, Seton RE, Steiner L, Stevick P, Suarez PL, Whooley P (2009). Current knowledge on the distribution and relative abundance of Humpback Whales (*Megaptera novaeangliae*) off the Cape Verde Islands, Eastern North Atlantic. Aquatic Mammals.

[ref-82] Wienerroither R, Uiblein F, Bordes F, Moreno T (2009). Composition, distribution, and diversity of pelagic fishes around the Canary Islands, Eastern Central Atlantic. Marine Biology Research.

[ref-83] Wood MJ (2007). Parasites entangled in food webs. Trends in Parasitology.

[ref-84] Wood CL, Micheli F, Fernández M, Gelcich S, Castilla JC, Carvajal J (2013). Marine protected areas facilitate parasite populations among four fished host species of central Chile. Journal of Animal Ecology.

[ref-85] Wood CL, Sandin SA, Zgliczynski B, Guerra AS, Micheli F (2014). Fishing drives declines in fish parasite diversity and has variable effects on parasite abundance. Ecology.

[ref-86] WoRMS Editorial Board (2017). World register of marine species. http://www.marinespecies.org.

[ref-87] Yatsu A, Sassa C, Moku M, Kinoshita T (2005). Night-time vertical distribution and abundance of small epipelagic and mesopelagic fishes in the upper 100 m layer of the Kuroshio-Oyashio Transition Zone in Spring. Fisheries Science.

[ref-88] Zhu X, Gasser RB, Jacobs DE, Hung GC, Chilton NB (2000). Relationships among some ascaridoid nematodes based on ribosomal DNA sequence data. Parasitology Research.

[ref-89] Zhu X, Gasser RB, Podolska M, Chilton NB (1998). Characterisation of anisakid nematodes with zoonotic potential by nuclear ribosomal dna sequences. International Journal for Parasitology.

