# Supplementary material for: Mesopredatory fishes from the subtropical upwelling region off NW-Africa characterised by their parasite fauna
Source: PeerJ. 2018 Aug 8;6:e5339. doi: 10.7717/peerj.5339 (PMC6087424; doi:10.7717/peerj.5339)
Supplement: Table S1 — ci, confidence interval; CW, carcass weight; GW, gonad weight; LW, liver weight; max, maximum; min, minimum; N.l., N. tripes; SD, standard deviation; SL, standard length; TL, total length; T.l., T. lepturus; TW, total weight. [file peerj-06-5339-s001.docx]

Supplemental Table S1: Morphometric measures of *Trichiurus lepturus* and *Nealotus tripes*. ci = confidence interval, CW = carcass weight, GW = gonad weight, LW = liver weight, max = maximum, min = minimum, *N.l.* = *N. tripes*, SD = standard deviation, SL = standard length, TL = total length, *T.l.* = *T. lepturus*, TW = total weight.

|  | SL [cm] | | TL [cm] | | PL [cm] | | TW [g] | | CW [g] | | GW [g] | | LW [g] | |
| --- | --- | --- | --- | --- | --- | --- | --- | --- | --- | --- | --- | --- | --- | --- |
|  | *T.l.* | *N.t.* | *T.l.* | *N.t.* | *T.l.* | *N.t.* | *T.l.* | *N.t.* | *T.l.* | *N.t.* | *T.l.* | N.t. | *T.l.* | N.t. |
| mean±SD | 59.4±6.7 | 16.5**±**1.1 | 60.3±6.4 | 18.0±1.3 | 21.2±2.4 | 11.2±0.8 | 146.6±56.3 | 24.3±6.2 | 134.9±52.4 | 21.5±5.3 | 0.33±0.45 | 0.38±0.65 | 0.92±0.43 | 0.29±0.18 |
| 95 % ci | 58.1−60.7 | 16.2−16.7 | 59.1−61.6 | 17.8−18.3 | 20.8−21.7 | 11.0−11.4 | 135.6−157.5 | 23.0−25.6 | 124.6−145.1 | 20.4−22.6 | 0.24−0.42 | 0.24−0.52 | 0.83−1.00 | 0.25−0.33 |
| median | 58.7 | 16.4 | 59.6 | 18.0 | 20.8 | 11.1 | 128.1 | 23.3 | 120.3 | 20.3 | 0.17 | 0.10 | 0.78 | 0.22 |
| min - max | 43.2−75.5 | 14.3−20.2 | 50.6−76.8 | 15.8−22.3 | 17.3−26.9 | 9.7−14.1 | 75.0−319.8 | 12.5−44.2 | 65.8−297.4 | 12.4−38.0 | 0.02−3.04 | 0.02−3.14 | 0.45−2.78 | 0.09−0.97 |
