# Supplementary material for: Mesopredatory fishes from the subtropical upwelling region off NW-Africa characterised by their parasite fauna
Source: PeerJ. 2018 Aug 8;6:e5339. doi: 10.7717/peerj.5339 (PMC6087424; doi:10.7717/peerj.5339)
Supplement: Table S2 — Reported parasites of T. lepturus, parasite taxa and prevalences (if available) from the literature with corresponding references. Asterisks indicate parasites discovered in the present study. BC, Brazil Current; CC, Canary Current; FC, Falkland Current; KC, Kuroshio Current; P, Prevalence; TWC, Taiwan Warm Current; ZMCC, Zhe-Min Coastal Current. [file peerj-06-5339-s002.docx]

Supplemental Table S2: Parasite fauna of *Trichiurus lepturus* according to online research. Reported parasites of *T. lepturus*, parasite taxa and prevalences (if available) from the literature with corresponding references. Asterisks indicate parasites discovered in the present study. BC = Brazil Current, CC = Canary Current, FC = Falkland Current, KC = Kuroshio Current, P = Prevalence, TWC = Taiwan Warm Current, ZMCC = Zhe-Min Coastal Current.

|  | P [%] | Current | Region | Reference |
| --- | --- | --- | --- | --- |
| **Myxozoa** |  |  |  |  |
| *Ceratomyxa trichiuri* |  | CC | Senegal | Kpatcha et al., 1996 |
| **Digenea** |  |  |  |  |
| *Aponurus megaloporus* |  |  | South China Sea | Shen & Li, 2000 |
| *Azygia longa* |  |  |  | Sillman, 1962 |
| *Bucephalus brevitentaculatus* |  |  | Barataria Bay, Louisiana | Corkum, 1967 |
| *Catarinatrema verrucosum* | 100.0 |  |  | Freitas & Santos, 1971; Cavalcanti et al., 2012 |
| *Ectenurus trichiuri* |  |  | East China Sea | Gu & Shen, 1981; Shen, 1990a |
| *Hirudinella ventricosa* |  |  |  | Linton, 1940 |
| *Lecithochirium acutum* |  |  | Persian Gulf | Gupta & Sehgal, 1971; Sey et al., 2003 |
| *L. branchiale* |  |  | Taiwanese Sea | Wang, 1982 |
| *L. excisum* |  | KC | Sea of Japan | Zhukov, 1960 |
| *L. ghanense* |  |  | West Africa | Fischthal, 1972 |
| *L. grandiporum* |  |  | South China Sea, Taiwanese Sea | Shen, 1990b; Liu, 1996 |
| *L. holocentri* |  |  | South China Sea, East China Sea | Gu & Shen, 1981; Shen, 1987 |
| *L. manteri* |  | BC | Guanabara | Freitas & Gomes, 1971 |
| *L. microstromum* * | 93.3 | BC, FC | Guanabara bay; Argentina, Uruguay | Freitas & Kohn, 1965; Silva et al., 2000; Silva, Luque & Ramos Alves, 2000; Carvalho & Luque, 2011 |
| *L. monticellii* |  |  |  | Nasir & Díaz, 1971 |
| *L. polynemi* |  |  |  | Gupta & Sehgal, 1971 |
| *L. savalae* |  |  | Taiwanese Sea | Shen, 1987 |
| *L. trichiuri* |  | KC | NE-Taiwan | Gu & Shen, 1981; Shen & Qiu, 1995; Shih, 2004 |
| *Lecithocladium pagrosomi* |  |  | South China Sea | Gu & Shen, 1981 |
| *L. parviovum* |  |  | South China Sea | Gu & Shen, 1981 |
| *Lepocreadium trullaforme* |  |  | Massachusetts | Linton, 1940 |
| *Musculovesicula trichiuri* |  |  |  | Gu & Shen, 1981 |
| *Opechona pyriforme* |  |  | NE-Atlantic | Bray & Gibson, 1990 |
| *Parahemiurus trichiuri* |  |  |  | Gu & Shen, 1981 |
| *Plerurus digitatus* |  |  | S-Indian Ocean Great Barrier Reef Philippines, Taiwanese Sea, South China Sea | Gu & Shen, 1981; Wang, 1987; Bray, 1990; Shen, 1990b; Bray, Cribb & Barker, 1993; Arthur & Lumanlan-Mayo, 1997 |
| *Prosorhynchoides arcuatus* |  |  | Massachusetts | Linton, 1940 |
| *Prosorhynchoides scombropis* |  |  |  | Huang, 2001; Liu et al., 2010 |
| *Prosorhynchoides trichiuri* |  |  |  | Sogandares-Bernal, 1955 |
| *Prosorhynchus manteri* |  |  | Arabian Gulf, Kuwait | Nahhas, Sey & Nakahara, 2006 |
| *Pseudopecoelus elongatus* |  | BC | Guanabara bay | Vicente & Santos, 1974; Carvalho & Luque, 2011 |
| *Pseudopecoelus tortugae* |  |  | Ghana | Veléz, 1987 |
| *Tubulovesicula angusticauda* |  |  | South China Sea | Gu & Shen, 1981; Shen, 1990b |
| *Tubulovesicula trichiuri* |  |  | South China Sea | Gu & Shen, 1978 |
| *Schikhobalotrema acuta* |  |  | Venezuela | Fischthal & Nasir, 1974 |
| **Monogenea** |  |  |  |  |
| Capsalidae gen. sp. | 1.7  2.0 | BC | Ubatuba | Rohde, Hayward & Heap, 1995; Bueno, Cenci de Aguiar & Santos, 2014 |
| *Diplectanotrema* sp. | 18.0 | BC | SE-Brazil | Silva et al., 2000; Carvalho & Luque, 2012 |
| *Encotyllabe souzalimae* | 7.5 | BC | SE-Brazil | Carvalho and Luque, 2012 |
| *Microcotyle* sp. | 3.3 | BC | Guanabara bay | Carvalho and Luque, 2011 |
| *Neobenedenia melleni* | 8.3 | BC | Guanabara bay | Carvalho and Luque, 2011 |
| *Octoplectanocotyla travassosi* * |  | BC | Guanabara bay  SE-Brazil | Carvalho and Luque, 2011  Carvalho and Luque, 2012 |
| *O. trichiuri* | 32,7 | BC | SE-Brazil | Sivasankara Pillai & Krishna Pillai 1978; Gajevskaja & Aljoshkina, 1988; Silva et al., 2000; Silva, Luque & Ramos Alves, 2000; Huang, 2001; Zhang, Jianying et al., 2003; Carvalho & Luque, 2012 |
| *Pseudempleurosoma guanabarensis* | 16.7 | BC | SE-Brazil | Rohde, Hayward & Heap, 1995; Carvalho and Luque, 2012 |
| **Cestoda** |  |  |  |  |
| *Callitetrarhynchus gracilis* | 12.5 | BC | Guanabara bay | Carvalho and Luque, 2011  Kardousha, 1999; Silva, Luque & Ramos Alves, 2000; Carvalho & Luque, 2011 |
| *Floriceps* sp. |  |  |  | El-Naffar et al., 1992 |
| *Nybelinia* sp. |  |  |  | Bates, 1990; El-Naffar et al., 1992 |
| *Nybelinia bisulcata* |  |  |  | Kardousha, 1999 |
| *Proteocephalus* spp. |  |  | NE-Taiwan | Shih, 2004 |
| *Prosobothrium* spp, |  |  |  | Avdeeva, 1989 |
| *Pterobothrium* sp. |  |  |  | El-Naffar et al., 1992 |
| *P. heteracanthum* |  |  |  | Kardousha, 1999 |
| *P. interruptum* |  |  |  | Campbell & Beveridge, 1996 |
| Trypanorhyncha indet. | 30.0 | BC | Ubatuba | Bueno et al., 2014 |
| Tetraphyllidea indet. | 65.0 | BC | Guanabara bay | Carvalho and Luque, 2011 |
|  | 86.0 | BC | Ubatuba | Bueno et al., 2014  Silva, 2000 |
| **Nematoda** |  |  |  |  |
| *Angusticaecum singhi* |  |  | Arabian Sea | Bruce, Adlard & Cannon, 1994 |
| *Anisakis* sp. |  |  |  | Silva, Luque & Ramos Alves, 2000 |
| *A. pegreffii* * | 84.4 | ZMCC | East China Sea | Kong et al., 2015 |
| *A. simplex* (*s.s.*) |  | KC | NE-Taiwan | Shih, 2004 |
| *A. simplex* (*s.l.*) |  |  | Persina Gulf | Sey & Petter, 1997 |
| *A. typica* * | 20.3 | BC | Guanbara bay | Borges et al., 2012 |
| *Camallanides* sp. |  |  | Persian Gulf | Sey & Petter, 1997 |
| *Camallanus carangis* |  |  | Philippines | Rigby, Adamson & Deardorff, 1998 |
| *Camallanus dollfusi* |  |  | Coast of Bangladesh | Arthur & Ahmed, 2002 |
| *Camallanus marinus* |  |  | Philippines | Arthur & Lumanlan-Mayo, 1997 |
| *Capillaria* sp. |  |  |  | Arthur & Ahmed, 2002 |
| *Contracaecum* sp. |  |  |  | Silva, Luque & Ramos Alves, 2000 |
| *Hysterothylacium* spp. * | 51.5 | BC | Guanbara bay  Persian Gulf | Kardousha, 1992; Sun, Koyama & Kagei, 1992; Sun, 1993; Sey & Petter, 1997; Cavalcanti et al., 2012; Borges et al., 2012 |
| *Hysterothylacium aduncum* |  | KC | NE-Taiwan | Shih, 2004 |
| *Philometroides trichiuri* |  |  | Java, Indonesia | Moravec, Walter & Yuniar, 2012 |
| *Procamallanus* sp*.* |  | BC | Rio de Janeiro | (Silva et al., 2000) |
| *Pseudoterranova spp.* |  |  |  | (Silva, Luque & Ramos Alves, 2000)  El-Naffar et al., 1992; Sun, 1993; Paraguassú, Luque & Alves, 2000 |
| *Pseudoterranova decipiens* (*s.l.*) |  | KC | NE-Taiwan | Shih, 2004 |
| *Pulchascaris secunda* |  |  |  | Bruce, Adlard & Cannon, 1994 |
| *Terranova* sp. |  |  | Persian Gulf | Sey & Petter, 1997, 1998 |
| *Terranova trichiuri* sp. inq. |  |  |  | Bruce, Adlard & Cannon, 1994 |
| *Raphidascaris* spp. |  |  |  | Silva, Luque & Ramos Alves, 2000 |
| *Raphidascaris trichiuri* |  | KC | NE-Taiwan | Shih, 2004  Huang, 2001 |
| *Thynnascaris* sp. |  |  |  | Ma et al., 1997 |
| **Acanthocephala** |  |  |  |  |
| *Polymorphus* sp. | 56.0 | BC | Ubatuba | Silva et al., 2000; Silva, Luque & Ramos Alves, 2000; Bueno, Aguiar & Santos, 2014 |
| *Bolbosoma scomberomori* |  |  | East China Sea | Huang, 2001 |
| **Crustacea** |  |  |  |  |
| *Bomolochus* sp. | 2.0 | BC | Ubatuba | Silva et al., 2000; Bueno, Aguiar & Santos, 2014 |
| *Metacaligus latus* | 14.0 | TWC | Strait of Taiwan | Ho & Lin, 2002 |
| *M. rufus* | 10.0 |  |  | Rohde, Hayward & Heap, 1995 |
| *M. trichiuri* | 83.3  60.0 | BC | Guanabara bay, Mar del Plata | Rohde, Hayward & Heap, 1995; Boxshall & Montú, 1997; Luque, Chaves & Cezara, 1998; Silva et al., 2000; Silva, Luque & Ramos Alves, 2000; Carvalho & Luque, 2011; Paula et al., 2012; Bueno, Aguiar & Santos, 2014 |

**References**

Arthur JR, Ahmed ATA. 2002. *Checklist of the Parasites of Fishes of Bangladesh*. Food & Agriculture Org.

Arthur JR, Lumanlan-Mayo S. 1997. *Checklist of the parasites of fishes of the Philippines.* Rome: FAO.

Avdeeva NV. 1989. On the generic belonging of three types of larvae of cestodes of the collective genus „Scolex”. *Parazitologiya* 23:351–355.

Bates RM. 1990. *A checklist of the Trypanorhyncha (Platyhelminthes: Cestoda) of the world (1935-1985).* National Museum of Wales.

Borges JN, Cunha LFG, Santos HLC, Monteiro-Neto C, Santos CP. 2012. Morphological and Molecular Diagnosis of Anisakid Nematode Larvae from Cutlassfish (*Trichiurus lepturus*) off the Coast of Rio de Janeiro, Brazil. *PLoS ONE* 7:e40447. DOI: 10.1371/journal.pone.0040447.

Boxshall GA, Montú MA. 1997. Copepods parasitic on Brazilian coastal fishes: a handbook. *Nauplius* 5:1–225.

Bray RA. 1990. Hemiuridae (Digenea) from marine fishes of the southern Indian Ocean: Dinurinae, Elytrophallinae, Glomericirrinae and Plerurinae. *Systematic Parasitology* 17:183–217.

Bray RA, Cribb T, Barker S. 1993. Hemiuridae (digenea) from Marine Fishes of the Great-Barrier-Reef, Queensland, Australia. *Systematic Parasitology* 25:37–62. DOI: 10.1007/BF00017000.

Bray RA, Gibson D. 1990. The Lepocreadiidae (digenea) of Fishes of the North-East Atlantic - Review of the Genera *Opechona* Looss, 1907 and *Prodistomum* Linton, 1910. *Systematic Parasitology* 15:159–202. DOI: 10.1007/BF00010135.

Bruce NL, Adlard RD, Cannon LRG. 1994. Synoptic checklist of ascaridoid parasites (Nematoda) from fish hosts. *Invertebrate Systematics* 8:583–674. DOI: 10.1071/it9940583.

Bueno GBF, Aguiar JCC, Santos SMC dos. 2014. Community structure of metazoan parasites of *Trichiurus lepturus* (Perciformes, Trichiuridae) from Ubatuba, Southwestern Atlantic Ocean,Brazil. *Acta Scientiarum. Biological Sciences* 36:357. DOI: 10.4025/actascibiolsci.v36i3.21908.

Campbell RA, Beveridge I. 1996. Revision of the family Pterobothriidae Pintner, 1931 (Cestoda : Trypanorhyncha). *Invertebrate Systematics* 10:617–662. DOI: 10.1071/it9960617.

Carvalho AR, Luque JL. 2011. Seasonal variation in metazoan parasites of *Trichiurus lepturus* (Perciformes: Trichiuridae) of Rio de Janeiro, Brazil. *Brazilian Journal of Biology* 71:771–782.

Carvalho AR, Luque JL. 2012. Three new species of monogeneans parasitic on Atlantic cutlassfish *Trichiurus lepturus* (Perciformes: Trichiuridae) from Southeastern Brazil. *Acta Scientiarum. Biological Sciences* 34. DOI: 10.4025/actascibiolsci.v34i3.10360.

Cavalcanti ETS, Takemoto RM, Alves LC, Chellappa S. 2012. First report of metazoan fish parasites with zoonotic potential in *Scomberomorus brasiliensis* and *Trichiurus lepturus* from the coastal waters of Rio Grande do Norte, Brazil. *Marine Biodiversity Records* 5. DOI: 10.1017/S1755267212000292.

Corkum KC. 1967. Bucephalidae (Trematoda) in Fishes of the Northern Gulf of Mexico: *Bucephalus* Baer, 1827. *Transactions of the American Microscopical Society* 86:44–49. DOI: 10.2307/3224423.

El-Naffar MKI., Gobashy A., El-Etreby SG., Kardousha MM. 1992. General survey of helminth parasite genera of Arabian Gulf fishes (Coasts of United Arab Emirates). *Arab Gulf Journal of Scientific Research* 10.

Fischthal JH. 1972. Zoogeography of digenetic trematodes from West African marine fishes. *Proceedings of the Helminthological Society of Washington* 39:192–203.

Fischthal JH, Nasir P. 1974. Some digenetic trematodes from freshwater and marine fishes of Venezuela. *Norwegian Journal of Zoology* 22:71–80.

Freitas JFT de, Gomes DC. 1971. Sôbre uma nova espécie do gênero *Lecithochirium* Luehe, 1901: (Trematoda, Hemiuroidea). *Memórias do Instituto Oswaldo Cruz* 69:107–113. DOI: 10.1590/S0074-02761971000100008.

Freitas JFT de, Kohn A. 1965. Nova espécie do gênero *Glomericirrus* Yamaguti, 1937 (Trematoda, Hemiuridae). *Memórias do Instituto Oswaldo Cruz* 63:229–235.

Freitas JFT de, Santos E dos. 1971. Nôvo trematódeo parasito de peixe marinho. *Memórias do Instituto Oswaldo Cruz* 69:79–81.

Gajevskaja AV, Aljoshkina LD. 1988. Fauna of monogenea of the south-east Atlantic, its ecological and geographical analysis. *Zoologicheskij zhurnal. Moscow* 57:325–330.

Gu C, Shen JW. 1978. Some dinurid trematodes (Subfamily Dinurinae Looss, 1907) from marine fishes of eonomic importance of China. *Acta Zoologica Sinica* 24:373–387.

Gu C, Shen JW. 1981. Digenetic trematodes of ribbonfish, *Trichiurus haumela* (Forskal) and their distribution in the fishing grounds of China seas. *Acta Zoologica Sinica* 1:9.

Gupta NK, Sehgal SK. 1971. Studies on some hemiurid trematodes from marine food fishes in India. *Research Bulletin of the Panjab University of Science* 21.

Ho J, Lin CL. 2002. New Species of *Metacaligus* (Caligidae, Copepoda) Parasitic on the Cutlassfish (*Trichiurus lepturus*) of Taiwan, with a Cladistic Analysis of the Family Caligidae. *Zoological Science* 19:1363–1375. DOI: 10.2108/zsj.19.1363.

Huang Z. 2001. *Marine Species and Their Distribution in China’s Seas*. Krieger Publishing Company.

Kardousha MM. 1992. Helminth parasite larvae collected from Arabian Gulf fish (coasts of the United Arab Emirates) (1) Anisakid larvae (Nematoda: Anisakidae). *Japanese Journal of Parasitology* 41:464–472.

Kardousha MM. 1999. Helminth parasite larvae collected from Arabian Gulf fish II. First record of some trypanorhynch cestodes from economically important fishes. *Arab Gulf Journal of Scientific Research* 17:255–276.

Kong Q, Fan L, Zhang J, Akao N, Dong K, Lou D, Ding J, Tong Q, Zheng B, Chen R, Ohta N, Lu S. 2015. Molecular identification of *Anisakis* and *Hysterothylacium* larvae in marine fishes from the East China Sea and the Pacific coast of central Japan. *International Journal of Food Microbiology* 199:1–7. DOI: 10.1016/j.ijfoodmicro.2015.01.007.

Kpatcha TK, Diebakate C, Faye N, Toguebaye BS. 1996. New species of Myxosporidia of the genus Ceratomyxa Thelohan, 1895 parasites of marine fishes of Senegal, West Africa. *Parasite-Journal De La Societe Francaise De Parasitologie* 3:223–228. DOI: 10.1051/parasite/1996033223.

Linton E. 1940. Trematodes from fishes mainly from the Woods Hole region, Massachusetts. *Proceedings of the United States National Museum* 88:1–172.

Liu S. 1996. Two new species of *Lecithochirium* Luke, 1901 (Trematodes: Hemiuridae). *Journal of Oceanography in Taiwan Strait* 15:393–398.

Liu S, Peng W, Gao P, Fu M, Wu H, Lu M, Gao J, Xiao J. 2010. Digenean parasites of Chinese marine fishes: a list of species, hosts and geographical distribution. *Systematic Parasitology* 75:1. DOI: 10.1007/s11230-009-9211-9.

Luque JL, Chaves ND, Cezara AD. 1998. Novos registros de copépodes cologóideos parasitos de peixes marinhos do Brasil. *Nauplius* 6:9–16.

Ma HW, Jiang TJ, Quan FS, Chen XG, Wang HD, Zhang YS, Cui MS, Zhi WH, Jiang DC. 1997. The infection status of anisakid larvae in marine fish and cephalopods from the Bohai Sea, China and their taxonomical consideration. *The Korean Journal of Parasitology* 35:19–24.

Moravec F, Walter T, Yuniar AT. 2012. Five new species of philometrid nematodes (Philometridae) from marine fishes off Java, Indonesia. *Folia Parasitologica* 59:115–130. DOI: 10.14411/fp.2012.017.

Nahhas F, Sey O, Nakahara G. 2006. Digenetic trematodes of marine fishes from the Arabian Gulf off the coast of Kuwait. Family Bucephalidae Poche, 1907, and the description of a new species. *Helminthologia* 43:147–157. DOI: 10.2478/s11687-006-0028-7.

Nasir P, Díaz MT. 1971. A revision of genus *Lecithochirium* Luehe, 1901, and redescription of *L. monticellii* (Linton, 1898) Skrjabin and Guschanskaja, 1955. *Rivista di Parassitologia* 32:27–36.

Paraguassú AR, Luque JL, Alves DR. 2000. Aspectos quantitativos do parasitismo por larvas de anisakídeos (Nematoda: Ascaridoidea: Anisakidae) no pargo, *Pagrus pagrus* (Osteichthyes: Sparidae) do litoral do estado do Rio de Janeiro, Brasil. *Contribuições Avulsas sobre História Natural do Brasil, Série Zoologia* 24:1–8.

Paula C, Elizabeth B, Julia A., Laura L., Alejandra R., Gustavo V., Tomás T. 2012. New records of parasitic copepods (Crustacea, Copepoda) from marine fishes in the Argentinean Sea. *Acta Parasitologica* 57:83–89. DOI: 10.2478/s11686-012-0003-z.

Rigby MC, Adamson ML, Deardorff TL. 1998. *Camallanus carangis* Olsen, 1954 (Nematoda: Camallanidae) reported from French Polynesia and Hawai’i with a redescription of the species. *The Journal of Parasitology* 84:158–162.

Rohde K, Hayward C, Heap M. 1995. Aspects of the ecology of metazoan ectoparasites of marine fishes. *International Journal for Parasitology* 25:945–970. DOI: 10.1016/0020-7519(95)00015-T.

Sey O, Nahhas FM, Uch S, Vang C. 2003. Digenetic trematodes from marine fishes off the coast of Kuwait, Arabian Gulf: Fellodistomidae and some smaller families, new host and geographic records. *Acta Zoologica Academiae Hungaricae* 49:179–200.

Sey O, Petter AJ. 1997. Nematode parasites of marine fishes from Kuwait, with a description of *Cucullanus trachinoti* n.sp. from *Trachinotus blochi*. *Zoosystema* 19:35–59.

Sey O, Petter AJ. 1998. Prevalence of ascaridoid larvae in Kuwaiti food fishes. *Kuwait Journal of Science and Engineering* 25:435–442.

Shen JW. 1987. Digenetic trematodes of fishes from the East China Sea II. Description of a new genus and seven new species of Hemiurida. *Studia Marina Sinica* 27:125–139.

Shen JW. 1990a. Digenetic trematodes of fishes from the Changjiang River estuary. *Studia Marina Sinica* 31:115–120.

Shen JW. 1990b. *Digenetic trematodes of marine fishes from Hainan Island*. Beijing: Science Press.

Shen JW, Li X-H. 2000. Addendum to the parasite digenetic trematodes from marine fishes in Hainan Island. *Studia Marina Sinica* 42:153–162.

Shen JW, Qiu ZZ. 1995. *Studies on the trematodes of fishes from the Yellow Sea and the Bo Hai Sea*. Beijing: Science Press.

Shih HH. 2004. Parasitic helminth fauna of the cutlass fish, *Trichiurus lepturus* L., and the differentiation of four anisakid nematode third-stage larvae by nuclear ribosomal DNA sequences. *Parasitology Research* 93. DOI: 10.1007/s00436-004-1095-7.

Sillman EI. 1962. The Life History of *Azygia longa* (Leidy 1851) (Trematoda: Digenea), and Notes on *A. acuminata* Goldberger 1911. *Transactions of the American Microscopical Society* 81:43–65. DOI: 10.2307/3223942.

Silva LO da, Luque JL, Alves DR, Paraguassú AR. 2000. Ecologia da comunidade de metazoários parasitos do peixe-espada *Trichiurus lepturus* Linnaeus (Osteichthyes, Trichiuridae) do litoral do estado do Rio de Janeiro, Brasil. *Revista Brasileira de Zoociências* 2.

Silva LO da, Luque JL, Ramos Alves D. 2000. Metazoários parasitos do peixe espada, *Trichiurus lepturus* (Osteichthyes: Trichiuridae) do litoral do estado do Rio de Janeiro, Brasil. *Parasitología al día* 24:97–101. DOI: 10.4067/S0716-07202000000300005.

Sivasankara Pillai V, Krishna Pillai N. 1978. Monogenean parasites of the marine fishes of the Kerala coast. II. *Aquatic Biology* 3:99–120.

Sogandares-Bernal F. 1955. Some Helminth Parasites of Fresh and Brackish Water Fishes from Louisiana and Panama. *The Journal of Parasitology* 41:587–594. DOI: 10.2307/3274140.

Sun SZ. 1993. Morphological and taxonomical studies on Anisakidae larvae found in marine fishes and squids of China. I. Yellow Sea and East Sea. *Acta Zoologica Sinica* 39:130–138.

Sun SZ, Koyama T, Kagei N. 1992. Morphological and taxonomical studies on Anisakidae larvae found in marine fishes of China, II, Gulf of Tong King. *Chinese Journal of Parasitology & Parasitic Diseases* 10:108–112.

Veléz IE. 1987. Sobre la fauna de trematodos en peces marinos de la familia Lutjanidae en el Mar Caribe. *Actualidades Biológicas* 16:70–84.

Vicente JJ, Santos E dos. 1974. Alguns Helmintos de peixes do litoral norte fluminense - II. *Memórias do Instituto Oswaldo Cruz* 72:173–180. DOI: 10.1590/S0074-02761974000200002.

Wang PQ. 1982. Hemiuroid trematodes of marine fishes from Fujian Province, China. *The Journal of Fujian Teachers University (Natural Science)* 2:67–80.

Wang YY. 1987. Digenetic trematodes (Hemiuridae) of marine fishes from Fujian Province. *Wuyi Science Journal* 7:165–179.

Jianying Z, Tingbao Y, L Lin, Xuejuan D. 2003. A list of monogeneans from Chinese marine fishes. *Systematic Parasitology* 54:111–130. DOI: 10.1023/A:1022581523683.

Zhukov EV. 1960. Endoparasitic worms of fish from the Sea of Japan and the shallow waters of the South Kuril Islands. *Endoparasitic worms of fish from the Sea of Japan and the shallow waters of the South Kuril Islands.* 28.
