# Supplementary material for: Mesopredatory fishes from the subtropical upwelling region off NW-Africa characterised by their parasite fauna
Source: PeerJ. 2018 Aug 8;6:e5339. doi: 10.7717/peerj.5339 (PMC6087424; doi:10.7717/peerj.5339)
Supplement: Data S1 — ID, host identification code, Hol., catch number; SL, standard lenght; TL, total length; PL, preanal length; TW, total weight; CW, carcass weight; GW, gonad weight; LW, liver weight; SW, stomach weight. [file peerj-06-5339-s003.docx]

Supplemental Raw Data S1: Raw data of morphometrics and diet of *Trichiurus lepturus* and *Nealotus tripes* specimens. ID = specimen code, Hol. = catch number, SL = standard lenght, TL = total length, PL = preanal length, TW = total weight, CW = carcass weight, GW = gonad weight, LW = liver weight, SW = stomach weight.

| ID | Hol. | Catch date | SL [cm] | TL [cm] | PL [cm] | TW [g] | CW [g] | Sex | GW [g] | LW [g] | SW [g] | SW empty [g] | Food items | Food weight [g] |
| --- | --- | --- | --- | --- | --- | --- | --- | --- | --- | --- | --- | --- | --- | --- |
| T.l1 | 550/49 | 29/06/14 | 56.3 | 57.1 | 20.1 | 105.63 | 98.61 | f | 0.74 | 0.153 | 3.547 | 0.863 | 606 Mysida | 1.695 |
| T.l2 | 550/49 | 29/06/14 | 63.9 | 64.1 | 23.4 | 165.78 | 156.21 | f | 0.813 | 0.219 | 3.385 | 1.23 | 7 Mysida  13 Teleostei | < 0,001  1.378 |
| T.l3 | 550/49 | 29/06/14 | 62.9 | 62.9 | 22 | 143.62 | 132.16 | m | 0.811 | 0.229 | 3.001 | 1.144 | 226 Mysida  2 Teleostei | 1.209  0.447 |
| T.l4 | 550/49 | 29/06/14 | 54.9 | 55.2 | 18.7 | 91.61 | 82.29 | f | 0.597 | 0.168 | 4.284 | 1.163 | 725Mysida | 1.692 |
| T.l5 | 550/49 | 29/06/14 | 73 | 73.7 | 26.3 | 263.65 | 244.51 | f | 2.17 | 1.169 | 5.89 | 2.195 | 13 Mysida  3 Teleostei  1 Cephalopoda | 0.078  2.75  6.122 |
| T.l6 | 550/49 | 29/06/14 | 55.5 | 55.7 | 20.3 | 110.6 | 104.43 | m | 0.79 | 0.167 | 1.456 | 0.87 | 137 Mysida | 0.468 |
| T.l7 | 550/49 | 29/06/14 | 58.5 | 58.5 | 20.9 | 133.13 | 123.44 | m | 0.862 | 0.197 | 2.067 | 0.859 | 239 Mysida | 0.784 |
| T.l8 | 550/49 | 29/06/14 | 57 | 57.5 | 20.4 | 113.58 | 104.06 | m | 0.674 | 0.157 | 4.517 | 0.946 | 433 Mysida  1 Teleostei | 1.82  1.534 |
| T.l9 | 550/49 | 29/06/14 | 54.2 | 54.7 | 19.1 | 110.05 | 100.92 | m | 0.794 | 0.12 | 4.49 | 0.93 | 678 Mysida | 3.324 |
| T.l10 | 550/49 | 29/06/14 | 55.5 | 55.5 | 19.6 | 102.85 | 94.53 | f | 0.593 | 0.123 | 3.691 | 0.592 | 416 Mysida  1 Teleostei | 2.359  0.557 |
| T.l11 | 550/49 | 29/06/14 | 52.1 | 52.9 | 18.8 | 92.1 | 84.59 | m | 0.536 | 0.126 | 3.905 | 0.873 | 740 Mysida | 2.627 |
| T.l12 | 550/49 | 29/06/14 | 59.8 | 60.2 | 21 | 132.11 | 123 | m | 0.969 | 0.25 | 2.05 | 0.895 | 162 Mysida  1Teleostei | 0.423  0.345 |
| T.l13 | 550/49 | 29/06/14 | 54.2 | 55.4 | 18.9 | 95.89 | 87.7 | m | 0.606 | 0.115 | 3.1 | 0.694 | 528 Mysida | 2.137 |
| T.l14 | 550/49 | 29/06/14 | 73.1 | 74.2 | 26.6 | 290.12 | 274.11 | m | 1.553 | 3.041 | 1.976 | 1.492 | 26 Mysida | 0.142 |
| T.l15 | 550/49 | 29/06/14 | 75.5 | 76.8 | 26.9 | 319.75 | 297.35 | m | 1.86 | 1.512 | 11.08 | 2.74 | 1 Mysida  1 Teleostei | 0.07  0.328 |
| T.l16 | 550/49 | 29/06/14 | 59.4 | 59.9 | 21.4 | 143.56 | 128.02 | m | 0.815 | 0.148 | 8.335 | 1.144 | 1 Clupeiformes | 6.514 |
| T.l17 | 550/49 | 29/06/14 | 68.7 | 69.6 | 25 | 236.44 | 199.69 | f | 1.14 | 0.3 | 25.338 | 1.923 | 143 Mysida  5 Clupeiformes | 0.132  19.989 |
| T.l18 | 550/49 | 29/06/14 | 62.7 | 62.7 | 21.8 | 149.02 | 139.7 | m | 0.831 | 0.191 | 2.184 | 1.026 | 222 Mysida | 0.903 |
| T.l19 | 550/49 | 29/06/14 | 51.5 | 52 | 18.7 | 89.27 | 81.63 | m | 0.508 | 0.077 | 3.884 | 0.695 | 696 Mysida | 2.917 |
| T.l20 | 550/49 | 29/06/14 | 55.2 | 56.5 | 19.9 | 113.49 | 104.26 | m | 0.694 | 0.085 | 4.324 | 0.897 | 678 Mysida | 3.368 |
| T.l21 | 550/49 | 29/06/14 | 58.5 | 59 | 20.8 | 119.34 | 110.46 | f | 0.906 | 0.09 | 2.356 | 0.735 | 431 Mysida | 1.264 |
| T.l22 | 550/49 | 29/06/14 | 60.2 | 61.4 | 21.1 | 125.67 | 114.76 | f | 0.813 | 0.213 | 3.287 | 0.948 | 593 Mysida | 1.953 |
| T.l23 | 550/49 | 29/06/14 | 53.3 | 53.3 | 19.5 | 110.5 | 103.63 | m | 0.671 | 0.205 | 1.755 | 0.712 | 98 Mysida | 0.913 |
| T.l24 | 550/49 | 29/06/14 | 57 | 57.5 | 20.1 | 120.77 | 112.25 | m | 0.779 | 0.087 | 2.854 | 0.729 | 325Mysida  1 Cephalopoda | 1.038  0.588 |
| T.l25 | 550/49 | 29/06/14 | 52.1 | 52.3 | 18.8 | 98.56 | 90.86 | m | 1.011 | 0.09 | 3.972 | 0.825 | 571 Mysida | 2.976 |
| T.l26 | 550/49 | 29/06/14 | 64.3 | 65.1 | 22.9 | 170.33 | 157.29 | f | 1.035 | 0.201 | 5.448 | 1.21 | 9 Mysida  3 Clupeiformes | 0.125  2.992 |
| T.l27 | 550/49 | 29/06/14 | 58 | 59.1 | 21.1 | 127.46 | 119.8 | f | 0.943 | 0.145 | 2.744 | 0.976 | 154 Mysida  1 Teleostei  1 Cephalopoda | 0.562  0.481  0.202 |
| T.l28 | 550/49 | 29/06/14 | 55.5 | 56.4 | 18.7 | 104.94 | 97.25 | m | 0.74 | 0.092 | 3.583 | 0.707 | 737 Mysida | 2.73 |
| T.l29 | 550/49 | 29/06/14 | 52 | 52.8 | 18.9 | 88.85 | 79.45 | m | 0.569 | 0.06 | 4.935 | 0.769 | 828 Mysida | 3.798 |
| T.l30 | 550/49 | 29/06/14 | 62 | 62.7 | 22.6 | 165.62 | 154.46 | m | 1.174 | 0.41 | 3.833 | 1.199 | 442 Mysida  1 Teleostei | 2.135  0.152 |
| T.l31 | 550/49 | 29/06/14 | 53.5 | 54.2 | 18.4 | 93.92 | 86.42 | f | 0.561 | 0.135 | 3.281 | 0.607 | 552 Mysida | 2.735 |
| T.l32 | 550/49 | 29/06/14 | 61.5 | 61.9 | 21.3 | 149.38 | 140.22 | m | 0.75 | 0.275 | 3.112 | 0.844 | 531 Mysida | 1.983 |
| T.l33 | 550/49 | 29/06/14 | 65 | 65.9 | 23.6 | 186.41 | 174.55 | m | 0.995 | 0.637 | 2.351 | 1.18 | 157 Mysida  1 Teleostei  1 Cephalopoda | 0.488  0.378  0.011 |
| T.l34 | 550/49 | 29/06/14 | 54.2 | 55 | 18.5 | 92.57 | 84.86 | m | 0.51 | 0.09 | 4.216 | 0.542 | 728 Mysida | 3.476 |
| T.l35 | 550/49 | 29/06/14 | 60.7 | 61.7 | 21.5 | 140.32 | 130.75 | m | 0.77 | 0.247 | 4.265 | 1.038 | 288 Mysida  1 Teleostei | 1.529  1.206 |
| T.l36 | 550/49 | 29/06/14 | 59.4 | 59.4 | 23.8 | 200.58 | 190.36 | f | 1.24 | 0.258 | 1.72 | 1.183 | 1 Teleostei  3 Cephalopoda | 0.087  0.055 |
| T.l37 | 550/49 | 29/06/14 | 54.1 | 55.2 | 20.3 | 110.47 | 102.1 | f | 0.642 | 0.19 | 2.219 | 0.963 | 316 Mysida | 1.196 |
| T.l38 | 550/49 | 29/06/14 | 61.7 | 62.2 | 21.3 | 130 | 120.8 | f | 0.522 | 0.159 | 3.496 | 0.995 | 553 Mysida | 1.855 |
| T.l39 | 550/49 | 29/06/14 | 50.5 | 51.3 | 17.9 | 88.19 | 80.58 | f | 0.806 | 0.171 | 3.477 | 0.729 | 588 Mysida  1 Trichiuridae | 2.235  0.379 |
| T.l40 | 550/49 | 29/06/14 | 60.4 | 59.9 | 20.8 | 135.55 | 126.13 | f | 0.831 | 0.121 | 2.505 | 0.969 | 84 Mysida  1 Teleostei | 0.582  0.453 |
| T.l41 | 550/49 | 29/06/14 | 56.2 | 56.5 | 19.6 | 113.4 | 102.05 | f | 0.885 | 0.188 | 4.236 | 1.181 | 186 Mysida  3 Teleostei | 0.671  1.342 |
| T.l42 | 550/49 | 29/06/14 | 50.5 | 50.9 | 18 | 78.11 | 69.5 | m | 0.562 | 0.068 | 4.03 | 0.68 | 462 Mysida  1 Teleostei | 2.092  1.031 |
| T.l43 | 550/49 | 29/06/14 | 67.9 | 68.4 | 24.5 | 212.51 | 199.98 | f | 1.751 | 0.894 | 3.72 | 1.578 | 330 Mysida  3 Teleostei | 1.549  0.296 |
| T.l44 | 550/49 | 29/06/14 | 65.6 | 66 | 22.5 | 172.71 | 162.41 | f | 1.096 | 0.24 | 4.755 | 1.247 | 89 Mysida  1 Teleostei | 0.219  1.72 |
| T.l45 | 550/49 | 29/06/14 | 53.5 | 53.9 | 18.1 | 96.17 | 87.33 | f | 0.68 | 0.12 | 3.622 | 0.612 | 667 Mysida | 2.869 |
| T.l46 | 550/49 | 29/06/14 | 70.9 | 70.9 | 26 | 251.04 | 235.65 | m | 1.353 | 0.725 | 4.064 | 1.51 | 115 Mysida  3 Teleostei | 0.555  1.838 |
| T.l47 | 550/49 | 29/06/14 | 69.2 | 70.2 | 24.9 | 219.54 | 202.95 | f | 1.632 | 0.77 | 1.932 | 1.417 | 1 Mysida  8 Crustacea  1 Teleostei | <0.001  <0.001  0.08 |
| T.l48 | 550/49 | 29/06/14 | 63.4 | 63.9 | 22.3 | 163.86 | 141.78 | m | 0.847 | 0.153 | 2.098 | 1.001 | 63 Mysida  1 Teleostei | 0.09  0.503 |
| T.l49 | 550/49 | 29/06/14 | 60 | 60.6 | 21.2 | 150.23 | 136.11 | m | 0.707 | 0.158 | 8.44 | 1.577 | 26 Mysida  2 Clupeiformes | <0.001  5.332 |
| T.l50 | 550/49 | 29/06/14 | 52.7 | 52.7 | 19.1 | 101.76 | 90.22 | m | 0.731 | 0.166 | 5.408 | 0.9 | 184 Mysida  3 Teleostei | 0.717  2.98 |
| T.l51 | 550/49 | 29/06/14 | 51.3 | 51.3 | 18.5 | 89.47 | 81.15 | f | 0.622 | 0.174 | 3.555 | 0.782 | 628 Mysida | 2.632 |
| T.l52 | 550/49 | 29/06/14 | 71.5 | 71.9 | 26.5 | 250.25 | 232.26 | f | 2.401 | 1.158 | 6.887 | 2.191 | 2 Clupeiformes  1 Cephalopoda | 3.812  0.03 |
| T.l53 | 550/49 | 29/06/14 | 54.6 | 55 | 19.5 | 98.52 | 91.54 | m | 0.484 | 0.093 | 3.925 | 0.836 | 633 Mysida | 2.665 |
| T.l54 | 550/49 | 29/06/14 | 58.9 | 59.4 | 20.5 | 126.49 | 118.63 | f | 0.783 | 0.178 | 3.541 | 0.687 | 37 Mysida  1 Decapoda  2 Teleostei | 0.08  0.159  2.11 |
| T.l55 | 550/49 | 29/06/14 | 58.6 | 59.4 | 20.6 | 127.23 | 120.9 | m | 0.745 | 0.123 | 1.45 | 0.787 | 133 Mysida | 0.497 |
| T.l56 | 550/49 | 29/06/14 | 71.3 | 72.2 | 25.2 | 250 | 221.99 | f | 1.431 | 1.219 | 19.09 | 2.623 | 1 Clupeiformes | 15.008 |
| T.l57 | 550/49 | 29/06/14 | 55.5 | 56.2 | 19.8 | 100.17 | 93.2 | f | 0.747 | 0.189 | 4.149 | 1.136 | 353 Mysida  1 Teleostei | 1.277  0.146 |
| T.l58 | 550/49 | 29/06/14 | 56.2 | 57.3 | 20.1 | 113.9 | 106.17 | m | 0.751 | 0.078 | 3.315 | 0.763 | 501 Mysida  1 Teleostei | 2.327  0.01 |
| T.l59 | 550/49 | 29/06/14 | 55.9 | 55.9 | 21.6 | 148.85 | 140 | f | 0.748 | 0.132 | 2.229 | 0.982 | 332 Mysida | 0.977 |
| T.l60 | 550/49 | 29/06/14 | 51.6 | 52.3 | 18.4 | 93.76 | 82.39 | f | 0.675 | 0.111 | 6.38 | 0.829 | 956 Mysida | 5.414 |
| T.l61 | 550/49 | 29/06/14 | 61.2 | 62 | 21.4 | 139.91 | 133.17 | f | 0.845 | 0.271 | 2.615 | 0.89 | 424 Mysida | 1.45 |
| T.l62 | 550/49 | 29/06/14 | 67.3 | 68.5 | 23 | 244.25 | 229.15 | f | 2.227 | 0.758 | 4.25 | 2.155 | 14 Mysida  1 Teleostei | < 0,001  1.562 |
| T.l63 | 550/49 | 29/06/14 | 54.6 | 55.8 | 19 | 100.82 | 92 | f | 0.562 | 0.155 | 5.287 | 1.067 | 472 Mysida  2 Teleostei | 2.286  1.49 |
| T.l64 | 550/49 | 29/06/14 | 49.8 | 50.8 | 17.8 | 83.79 | 73.69 | m | 0.488 | 0.46 | 5.6 | 0.782 | 769 Mysida | 4.549 |
| T.l65 | 550/49 | 29/06/14 | 66.4 | 66.4 | 24.9 | 196.34 | 186.69 | f | 0.984 | 0.433 | 2.223 | 1.458 | 11 Mysida  1 Teleostei  1 Cephalopoda | 0.035  0.278  0.076 |
| T.l66 | 550/49 | 29/06/14 | 50.3 | 50.6 | 18.1 | 82.17 | 73.83 | n/a | 0.577 | 0.094 | 3.355 | 0.74 | 516 Mysida | 2.302 |
| T.l67 | 550/49 | 29/06/14 | 58.7 | 59.2 | 20.4 | 120.89 | 112.33 | f | 0.736 | 0.131 | 4.528 | 0.866 | 687 Mysida | 3.458 |
| T.l68 | 550/49 | 29/06/14 | 61 | 61.9 | 21.9 | 144.81 | 132.4 | f | 0.742 | 0.312 | 2.566 | 0.99 | 331 Mysida  1 Teleostei | 1.439  0.01 |
| T.l69 | 550/49 | 29/06/14 | 65.6 | 66.3 | 23.5 | 192.55 | 177.67 | f | 0.965 | 0.372 | 6.59 | 1.399 | 52 Mysida  3 Clupeiformes | < 0,001  4.408 |
| T.l70 | 550/49 | 29/06/14 | 61.5 | 61.9 | 21.9 | 142.44 | 135.19 | f | 0.728 | 0.351 | 2.009 | 1.003 | 191 Mysida | 0.702 |
| T.l71 | 550/49 | 29/06/14 | 49.8 | 54.3 | 19.3 | 99.65 | 86.15 | f | 0.536 | 0.14 | 3.82 | ... | 449 Mysida | / |
| T.l72 | 550/49 | 29/06/14 | 54.3 | 58 | 20.2 | 118.28 | 95.8 | m | 0.52 | 0.153 | 3.185 | 0.786 | 598 Mysida | 2.182 |
| T.l73 | 550/49 | 29/06/14 | 53 | 53.8 | 19.1 | 110.66 | 94.31 | m | 0.639 | 0.113 | 9.083 |  | 335 Mysida  2 Teleostei  1 Cephalopoda | 2.733  2.09  1.508 |
| T.l74 | 550/49 | 29/06/14 | 57.8 | 57.8 | 20.2 | 124.53 | 114.67 | m | 0.703 | 0.128 | 3.871 | 0.791 | 614 Mysida | 2.342 |
| T.l75 | 550/49 | 29/06/14 | 62 | 64.5 | 22.7 | 163.33 | 155.63 | m | 0.753 | 0.222 | 2.66 | 1.19 | 285 Mysida | 0.92 |
| T.l76 | 550/49 | 29/06/14 | 62.3 | 62.3 | 22 | 139.67 | 131.77 | m | 0.603 | 0.131 | 2.179 | 0.766 | 232 Mysida  1 Teleostei | 1.176  0.015 |
| T.l77 | 550/49 | 29/06/14 | 74.2 | 75.2 | 26.3 | 287.34 | 268.12 | m | 1.758 | 2.251 | 4.344 | 2.16 | 2 Mysida  1 Teleostei | ???  2.136 |
| T.l78 | 550/49 | 29/06/14 | 67 | 68 | 23.5 | 190.13 | 178.15 | m | 0.871 | 0.288 | 1.446 | 1.187 | 54 Mysida | 0.259 |
| T.l79 | 550/49 | 29/06/14 | 59.8 | 61.5 | 21 | 127.54 | 118.21 | m | 0.653 | 0.156 | 3.181 | 0.79 | 611 Mysida | 2.325 |
| T.l80 | 550/49 | 29/06/14 | 66.2 | 66.2 | 22.8 | 186.77 | 174.53 | m | 1.71 | 0.532 | 1.513 | 1.169 | 60 Mysida  2 Teleostei | 0.186  0.072 |
| T.l81 | 550/49 | 29/06/14 | 74 | 74 | 26.4 | 305.65 | 285.12 | f | 2.787 | 1.715 | 3.48 | 2.294 | 2 Teleostei | 2.392 |
| T.l82 | 550/49 | 29/06/14 | 53.3 | 53.8 | 18.5 | 95.94 | 88.95 | f | 0.617 | 0.082 | 2.176 | 0.629 | 420 Mysida | 1.48 |
| T.l83 | 550/49 | 29/06/14 | 67.5 | 69 | 23.5 | 182.37 | 171.73 | m | 0.801 | 0.525 | 2.445 | 1.218 | 307 Mysida | 1.147 |
| T.l84 | 550/49 | 29/06/14 | 67.7 | 68.2 | 23.7 | 211.86 | 176.77 | m | 1.195 | 0.452 | 14.987 | 2.07 | 43 Mysida  2 Clupeiformes | 0.622  10.445 |
| T.l85 | 550/49 | 29/06/14 | 58.8 | 61.3 | 21.4 | 142.28 | 127.98 | m | 0.858 | 0.155 | 6.286 | 1.468 | 215 Mysida  3 Clupeiformes | 0.702  4.942 |
| T.l86 | 550/49 | 29/06/14 | 43.2 | 64.3 | 22.7 | 171.73 | 154.39 | m | 1.29 | 0.248 | 9.782 | 1.532 | 102 Mysida  2 Clupeiformes | 0.734  8.128 |
| T.l87 | 550/49 | 29/06/14 | 49.1 | 51 | 17.3 | 75.07 | 65.82 | m | 0.456 | 0.049 | 2.341 | 0.503 | 322 Mysida | 1.816 |
| T.l88 | 550/49 | 29/06/14 | 65.5 | 65.5 | 22.5 | 172.92 | 161.24 | m | 0.896 | 0.25 | 3.209 | 1.105 | 429 Mysida | 2.065 |
| T.l89 | 550/49 | 29/06/14 | 55.7 | 56.8 | 19.5 | 102.35 | 93.54 | m | 0.586 | 0.149 | 3.449 | 0.649 | 582 Mysida | 2.715 |
| T.l90 | 550/49 | 29/06/14 | 54.2 | 54.2 | 19.5 | 109.29 | 102.04 | m | 1.092 | 0.225 | 0.973 | 0.973 |  |  |
| T.l91 | 550/49 | 29/06/14 | 56.2 | 57.5 | 20 | 123.63 | 114.34 | m | 0.727 | 0.125 | 3.829 | 0.918 | 562 Mysida  1 Teleostei | 2.575  0.102 |
| T.l92 | 550/49 | 29/06/14 | 58.1 | 61 | 20.5 | 137.6 | 124.87 | m | 0.962 | 0.105 | 7.131 | 0.992 | 260 Mysida  2 Clupeiformes | 1.053  4.066 |
| T.l93 | 550/49 | 29/06/14 | 69.2 | 69.2 | 24.7 | 242.79 | 219.1 | f | 1.769 | 0.532 | 13.462 | 1.726 | 1 Clupeiformes | 10.905 |
| T.l94 | 550/49 | 29/06/14 | 49.5 | 51.7 | 18.5 | 85.98 | 76.75 | m | 0.504 | 0.029 | 5.733 | 0.79 | 805 Mysida | 4.737 |
| T.l95 | 550/49 | 29/06/14 | 65 | 67.1 | 23.6 | 235.61 | 209.73 | m | 1.14 | 1.023 | 16.64 | 1.675 | 108 Mysida  3 Clupeiformes | 0.075  12.838 |
| T.l96 | 550/49 | 29/06/14 | 62.6 | 62.6 | 22.5 | 180.7 | 173.48 | m | 1.027 | 0.302 | 6.6 | 1.36 | 4 Teleostei | 5.86 |
| T.l97 | 550/49 | 29/06/14 | 59.8 | 59.8 | 26 | 128.64 | 118.99 | m | 0.783 | 0.174 | 2.775 | 0.708 | 205 Mysida  1 Teleostei | 0.979  0.764 |
| T.l98 | 550/49 | 29/06/14 | 67 | 67 | 24.5 | 246.64 | 215.72 | m | 1.173 | 0.803 | 22.82 | 1598 | 1 Clupeiformes | 20.449 |
| T.l99 | 550/49 | 29/06/14 | 67.4 | 67.7 | 23.6 | 199.6 | 190.27 | f | 1.592 | 0.401 | 2.584 | 1.381 | 81 Mysida  2 Teleostei | 0.24  0.462 |
| T.l100 | 550/49 | 29/06/14 | 59.4 | 61.6 | 20.6 | 152.13 | 142.63 | m | 1.207 | 0.321 | 4.067 | 0.91 | 307 Mysida  1Teleostei | 1.615  1.147 |
| T.l101 | 550/49 | 29/06/14 | 49.5 | 52 | 17.7 | 76.01 | 69.38 | f | 0.496 | 0.058 | 2.47 | 0.38 | 314 Mysida  1 Teleostei | 1.695  0.285 |
| T.l102 | 550/49 | 29/06/14 | 58.5 | 58.9 | 20.1 | 125.37 | 117.73 | f | 0.712 | 0.176 | 1.912 | 0.826 | 235 Mysida | 0.892 |
| T.l103 | 550/49 | 29/06/14 | 59.5 | 60.5 | 20.6 | 124.51 | 115.26 | m | 0.816 | 0.112 | 3.847 | 0.916 | 683 Mysida | 2.97 |
| T.l104 | 550/49 | 29/06/14 | 52.8 | 54 | 18 | 97.54 | 90.78 | f | 0.662 | 0.125 | 2.205 | 0.528 | 362 Mysida | 1.029 |
| N.t1 | 558/57 | 05/07/14 | 15.3 | 16.7 | 10.3 | 14.95 | 14.09 | m | 0.049 | 0.104 | 0.239 | 0.112 | 1 Teleostei | 0.11 |
| N.t2 | 558/57 | 05/07/14 | 18.1 | 19.9 | 12.5 | 33.37 | 29.27 | m | 0.129 | 0.296 | 0.543 | 0.361 | 1 Teleostei | 0.119 |
| N.t3 | 558/57 | 05/07/14 | 16.1 | 17.4 | 11.2 | 24.42 | 20.39 | f | 0.199 | 0.278 | 2.39 | 0.29 | 2 Myctophidae | 1.922 |
| N.t4 | 558/57 | 05/07/14 | 16.7 | 18 | 11.3 | 22.31 | 19.98 | m | 0.077 | 0.205 | 0.944 | 0.169 | 1 Teleostei | 0.628 |
| N.t5 | 558/57 | 05/07/14 | 17.3 | 18.9 | 11.9 | 29.52 | 26.63 | m | 0.226 | 0.339 | 1.574 | 0.303 | 2 Teleostei | 1.206 |
| N.t6 | 558/57 | 05/07/14 | 15.1 | 17 | 10.3 | 21.88 | 17.2 | f | 0.07 | 0.237 | 3.491 | 0.285 | 1 Myctophidae | 3.113 |
| N.t7 | 558/57 | 05/07/14 | 16.1 | 17.3 | 10.5 | 21.42 | 19.01 | m | 0.064 | 0.18 | 1.318 | 0.194 | 1 Teleostei  1 Decapoda | 0.897  0.196 |
| N.t8 | 558/57 | 05/07/14 | 15.7 | 17.1 | 10.6 | 20.2 | 17.88 | f | 0.171 | 0.207 | 0.943 | 0.166 | 1 Teleostei | 0.734 |
| N.t9 | 558/57 | 05/07/14 | 14.3 | 16.1 | 9.8 | 16.67 | 13.44 | f | 0.035 | 0.207 | 2.221 | 0.159 | 2 Myctophidae | 2.036 |
| N.t10 | 558/57 | 05/07/14 | 15.5 | 16.8 | 10.7 | 16.52 | 15.37 | m | 0.043 | 0.143 | 0.33 | 0.134 | 1 Teleostei | 0.173 |
| N.t11 | 558/57 | 05/07/14 | 17.2 | 18.6 | 11.9 | 27.69 | 24.49 | f | 0.719 | 0.394 | 0.988 | 0.307 | 1 Teleostei | 0.602 |
| N.t12 | 558/57 | 05/07/14 | 16.9 | 19.3 | 11.5 | 26.91 | 23.31 | f | 1.897 | 0.371 | 0.44 | 0.214 | 1 Teleostei | 0.22 |
| N.t13 | 558/57 | 05/07/14 | 15.6 | 17.2 | 10.8 | 18.86 | 17.87 | m | 0.084 | 0.139 | 0.429 | 0.139 | 1 Teleostei | 0.208 |
| N.t14 | 558/57 | 05/07/14 | 17.1 | 18.6 | 11.6 | 26.78 | 23.71 | f | 0.266 | 0.268 | 1.532 | 0.234 | 1 Myctophidae  1 Cephalopoda | 1.209  <0.001 |
| N.t15 | 558/57 | 05/07/14 | 16.2 | 17.4 | 11 | 20.66 | 19.43 | f | 0.121 | 0.224 | 0.341 | 0.187 | 1 Teleostei | 0.09 |
| N.t16 | 558/57 | 05/07/14 | 16.7 | 18.8 | 11.5 | 24.82 | 22.82 | j | 0.028 | 0.344 | 0.868 | 0.486 | 1 Teleostei | 0.298 |
| N.t17 | 558/57 | 05/07/14 | 15.1 | 16.4 | 10.4 | 18.34 | 17.11 | m | 0.074 | 0.135 | 0.291 | 0.118 | 1 Teleostei | 0.133 |
| N.t18 | 558/57 | 05/07/14 | 16.3 | 18.5 | 13 | 22.72 | 19.59 | f | 0.049 | 0.227 | 2.063 | 0.212 | 1 Myctophidae | 1.832 |
| N.t19 | 558/57 | 05/07/14 | 17.6 | 19.5 | 12 | 27.85 | 24.48 | f | 1.04 | 0.545 | 1.526 | 0.261 | 2 Teleostei | 1.158 |
| N.t20 | 558/57 | 05/07/14 | 15.8 | 16.6 | 10.9 | 19.42 | 17.5 | m | 0.052 | 0.145 | 0.713 | 0.179 | 2 Teleostei | 0.491 |
| N.t21 | 558/57 | 05/07/14 | 17 | 18.5 | 11.5 | 26.57 | 24.32 | f | 0.295 | 0.338 | 0.512 | 0.276 | 1 Teleostei | 0.18 |
| N.t22 | 558/57 | 05/07/14 | 16.7 | 18.6 | 11.1 | 23.36 | 21.65 | f | 0.123 | 0.228 | 0.866 | 0.2 | 2 Teleostei  1 Decapoda | 0.434  0.187 |
| N.t23 | 558/57 | 05/07/14 | 16.6 | 18.4 | 11.7 | 23.48 | 19.69 | f | 0.055 | 0.172 | 1.527 | 0.255 | 1 Myctophidae | 1.182 |
| N.t24 | 558/57 | 05/07/14 | 18.9 | 20.3 | 12.8 | 34.98 | 30.24 | f | 2.455 | 0.751 | 0.673 | 0.233 | 1 Teleostei | 0.26 |
| N.t25 | 558/57 | 05/07/14 | 17 | 18.6 | 11.8 | 26.8 | 24.35 | f | 0.396 | 0.323 | 1.067 | 0.276 | 1 Teleostei | 0.755 |
| N.t26 | 558/57 | 05/07/14 | 15.8 | 16.9 | 10.7 | 19.8 | 17.4 | m | 0.055 | 0.212 | 2.163 | 0.185 | 3 Teleostei | 1.909 |
| N.t27 | 558/57 | 05/07/14 | 16 | 17.4 | 11 | 21.49 | 18.5 | f | 0.68 | 0.213 | 1.636 | 0.191 | 1 Myctophidae  1 Cephalopoda | 1.055  0.377 |
| N.t28 | 558/57 | 05/07/14 | 16.5 | 17.8 | 11.2 | 20.88 | 19.81 | f | 0.15 | 0.229 | 0.722 | 0.226 | 1 Teleostei | 0.447 |
| N.t29 | 558/57 | 05/07/14 | 14.5 | 15.9 | 9.9 | 14.35 | 13.23 | m | 0.027 | 0.13 | 0.279 | 0.126 | 1 Teleostei | 0.128 |
| N.t30 | 558/57 | 05/07/14 | 16.3 | 18.9 | 11.1 | 25 | 18.7 | f | 0.066 | 0.158 | 4.945 | 0.264 | 2 Teleostei | 4.498 |
| N.t31 | 558/57 | 05/07/14 | 16.6 | 17.9 | 11.1 | 23.76 | 22.01 | m | 0.082 | 0.185 | 0.5 | 0.233 | 1 Teleostei | 0.249 |
| N.t32 | 558/57 | 05/07/14 | 20.1 | 22.1 | 13.2 | 41.99 | 35.6 | f | 2.448 | 0.977 | 1.384 | 0.379 | 1 Teleostei | 0.986 |
| N.t33 | 558/57 | 05/07/14 | 18.5 | 19.5 | 12.3 | 32.69 | 28.03 | f | 1.302 | 0.524 | 1.602 | 0.267 | 1 Myctophidae | 1.161 |
| N.t34 | 558/57 | 05/07/14 | 19.4 | 21.9 | 13.2 | 41.95 | 35.82 | f | 2.793 | 0.853 | 0.547 | 0.331 | 1 Teleostei | 0.201 |
| N.t35 | 558/57 | 05/07/14 | 15.5 | 17.2 | 10.4 | 18.96 | 16.18 | f | 0.039 | 0.233 | 1.875 | 0.19 | 1 Myctophidae | 1.586 |
| N.t36 | 558/57 | 05/07/14 | 17.8 | 19.4 | 11.9 | 29.54 | 26.62 | f | 1.146 | 0.532 | 0.977 | 0.231 | 1 Teleostei | 0.596 |
| N.t37 | 558/57 | 05/07/14 | 16.5 | 18.1 | 10.9 | 20.09 | 18.86 | n/a | 0.067 | 0.2 | 0.367 | 0.207 | 1 Teleostei | 0.143 |
| N.t38 | 558/57 | 05/07/14 | 16.4 | 18.5 | 11.1 | 26.98 | 24.2 | m | 0.285 | 0.367 | 0.959 | 0.274 | 1 Teleostei | 0.687 |
| N.t39 | 558/57 | 05/07/14 | 14.8 | 16.1 | 9.7 | 14.52 | 13.74 | m | 0.026 | 0.105 | 0.298 | 0.121 | 1 Teleostei | 0.128 |
| N.t40 | 558/57 | 05/07/14 | 16 | 17.6 | 11.2 | 20.05 | 17.84 | f | 0.163 | 0.35 | 1.067 | 0.189 | 2 Teleostei | 0.569 |
| N.t41 | 558/57 | 05/07/14 | 15.8 | 17.2 | 10.9 | 19.65 | 18.38 | m | 0.092 | 0.147 | 0.264 | 0.16 | 1 Teleostei | 0.025 |
| N.t42 | 558/57 | 05/07/14 | 16.2 | 17.9 | 11 | 21.48 | 19.71 | f | 0.064 | 0.22 | 0.402 | 0.279 | 1 Teleostei | 0.033 |
| N.t43 | 558/57 | 05/07/14 | 20.2 | 22.3 | 14.1 | 44.26 | 38.08 | f | 3.147 | 0.968 | 1.019 | 0.361 | 2 Teleostei | 0.53 |
| N.t44 | 558/57 | 05/07/14 | 15.9 | 16.8 | 10.5 | 23.59 | 19.58 | m | 0.058 | 0.168 | 2.679 | 0.238 | 1 Myctophidae | 2.315 |
| N.t45 | 558/57 | 05/07/14 | 17.2 | 18.7 | 11.5 | 25.52 | 23.46 | m | 0.1 | 0.202 | 0.642 | 0.225 | 1 Teleostei  1 Decapoda | 0.37  <0.001 |
| N.t46 | 558/57 | 05/07/14 | 18.1 | 19.7 | 12.2 | 33.19 | 30.36 | m | 0.147 | 0.28 | 0.794 | 0.326 | 1 Teleostei | 0.372 |
| N.t47 | 558/57 | 05/07/14 | 16.1 | 17.4 | 10.9 | 19.71 | 17.86 | f | 0.059 | 0.181 | 0.778 | 0.199 | 1 Teleostei | 0.352 |
| N.t48 | 558/57 | 05/07/14 | 18 | 19.6 | 11.8 | 29.32 | 25.86 | f | 1.26 | 0.485 | 0.938 | 0.337 | 1 Teleostei | 0.343 |
| N.t49 | 558/57 | 05/07/14 | 15.5 | 17.3 | 10.4 | 16.64 | 15.39 | f | 0.053 | 0.154 | 0.53 | 0.193 | 1 Teleostei | 0.25 |
| N.t50 | 558/57 | 05/07/14 | 16.2 | 18.3 | 11 | 22.65 | 19.6 | f | 0.107 | 0.176 | 1.484 | 0.161 | 1 Myctophidae | 1.115 |
| N.t51 | 558/57 | 05/07/14 | 16.2 | 18.5 | 10.9 | 23.62 | 20.55 | m | 0.076 | 0.223 | 1.542 | 0.221 | 1 Myctophidae | 1.172 |
| N.t52 | 558/57 | 05/07/14 | 15.6 | 17 | 10.4 | 17.38 | 14.78 | f | 0.037 | 0.098 | 1.324 | 0.173 | 1 Myctophidae  1 Decapoda | 1.026  0.026 |
| N.t53 | 558/57 | 05/07/14 | 15.1 | 16.8 | 10.2 | 17.42 | 15.04 | m | 0.037 | 0.226 | 0.571 | 0.127 | 2 Teleostei | 0.396 |
| N.t54 | 558/57 | 05/07/14 | 16.9 | 18.6 | 11.5 | 23.61 | 20.98 | f | 0.143 | 0.207 | 1.12 | 0.287 | 1 Teleostei | 0.64 |
| N.t55 | 558/57 | 05/07/14 | 14.5 | 15.9 | 10 | 14.31 | 12.47 | m | 0.025 | 0.094 | 1.11 | 0.121 | 1 Teleostei | 0.915 |
| N.t56 | 558/57 | 05/07/14 | 16.2 | 17.2 | 11 | 21.64 | 19.88 | f | 0.106 | 0.236 | 0.438 | 0.304 | 1 Teleostei | 0.079 |
| N.t57 | 558/57 | 05/07/14 | 16.6 | 17.9 | 10.9 | 22.52 | 21.27 | m | 0.091 | 0.148 | 0.764 | 0.237 | 1 Teleostei  1 Amphipoda | 0.477  <0.001 |
| N.t58 | 558/57 | 05/07/14 | 17.6 | 18.9 | 11.8 | 31 | 26.38 | f | 1.167 | 0.612 | 1.731 | 0.225 | 1 Myctophidae | 1.429 |
| N.t59 | 558/57 | 05/07/14 | 18.6 | 20.6 | 13 | 31.94 | 27.68 | f | 1.137 | 0.676 | 0.517 | 0.191 | 1 Teleostei | 0.245 |
| N.t60 | 558/57 | 05/07/14 | 17 | 19.1 | 11.5 | 25.34 | 21.65 | f | 0.25 | 0.255 | 1.74 | 0.18 | 3 Teleostei | 1.49 |
| N.t61 | 558/57 | 05/07/14 | 16.9 | 18.7 | 11.7 | 28.22 | 23.72 | f | 0.26 | 0.42 | 1.78 | 0.25 | 2 Teleostei | 1.44 |
| N.t62 | 558/57 | 05/07/14 | 16.2 | 18.1 | 11.1 | 22.1 | 19.8 | f | 0.02 | 0.18 | 0.9 | 0.2 | 1 Teleostei | 0.6 |
| N.t63 | 558/57 | 05/07/14 | 15.7 | 17.3 | 10.6 | 17.35 | 16.17 | f | 0.03 | 0.1 | 0.27 | 0.13 | 1 Teleostei | 0.06 |
| N.t64 | 558/57 | 05/07/14 | 16.4 | 17.8 | 11 | 22.85 | 20.5 | f | 0.1 | 0.26 | 0.97 | 0.18 | 1 Teleostei | 0.74 |
| N.t65 | 558/57 | 05/07/14 | 17.8 | 18.9 | 11.5 | 29.9 | 26.48 | f | 0.56 | 0.39 | 1.5 | 0.25 | 1 Teleostei | 1 |
| N.t66 | 558/57 | 05/07/14 | 16.4 | 18.5 | 11.5 | 23.31 | 20.22 | f | 0.02 | 0.22 | 1.71 | 0.2 | 2 Teleostei | 1.43 |
| N.t67 | 558/57 | 05/07/14 | 16.6 | 17.6 | 11.1 | 22.35 | 18.84 | f | 0.07 | 0.18 | 1.76 | 0.172 | 1 Teleostei | 1.3 |
| N.t68 | 558/57 | 05/07/14 | 15.8 | 17.3 | 10.6 | 21.4 | 18.63 | f | 0.09 | 0.13 | 1.05 | 0.15 | 1 Teleostei | 0.82 |
| N.t69 | 558/57 | 05/07/14 | 15.3 | 16.5 | 10.9 | 17.5 | 15.24 | f | 0.043 | 0.18 | 1.32 | 0.13 | 2 Teleostei | 1.1 |
| N.t70 | 558/57 | 05/07/14 | 17.3 | 18.5 | 11.6 | 27.7 | 24.78 | f | 0.125 | 0.36 | 0.73 | 0.22 | 2 Teleostei | 0.17 |
| N.t71 | 558/57 | 05/07/14 | 17 | 18.5 | 11.6 | 28.1 | 24.1 | f | 0.82 | 0.49 | 1.2 | 0.22 | 1 Teleostei | 0.8 |
| N.t72 | 558/57 | 05/07/14 | 16.4 | 18.1 | 11 | 25.4 | 23.03 | f | 0.29 | 0.24 | 0.41 | 0.18 | 1 Teleostei | 0.04 |
| N.t73 | 558/57 | 05/07/14 | 16.6 | 17.3 | 11.2 | 24.2 | 22.14 | f | 0.09 | 0.3 | 0.88 | 0.24 | 1 Teleostei | 0.26 |
| N.t74 | 558/57 | 05/07/14 | 17.9 | 18.9 | 12.4 | 32.1 | 29.2 | m | 0.09 | 0.35 | 0.93 | 0.28 | 1 Teleostei | 0.5 |
| N.t75 | 558/57 | 05/07/14 | 17.6 | 19.3 | 12.1 | 31.64 | 27.48 | f | 1.2 | 0.42 | 1.13 | 0.213 | 2 Teleostei | 0.85 |
| N.t76 | 558/57 | 05/07/14 | 16.7 | 17.4 | 11.3 | 28.73 | 24.17 | f | 0.53 | 0.44 | 1.96 | 0.197 | 2 Teleostei | 1.6 |
| N.t77 | 558/57 | 05/07/14 | 16.6 | 17.3 | 11.7 | 26.18 | 22.43 | f | 1.04 | 0.37 | 0.945 | 0.159 | 1 Teleostei | 0.64 |
| N.t78 | 558/57 | 05/07/14 | 17 | 18 | 11.5 | 24.55 | 22.27 | f | 0.09 | 0.21 | 0.54 | 0.18 | 1 Teleostei | 0.27 |
| N.t79 | 558/57 | 05/07/14 | 16.3 | 17.3 | 11 | 23.16 | 21.41 | m | 0.127 | 0.163 | 0.289 | 0.136 | 1 Teleostei | 0.07 |
| N.t80 | 558/57 | 05/07/14 | 15.7 | 16.8 | 11 | 21.45 | 18.97 | f | 0.101 | 0.248 | 0.807 | 0.168 | 1 Teleostei | 0.65 |
| N.t81 | 558/57 | 05/07/14 | 15.4 | 16.6 | 10.5 | 20.89 | 18.15 | m | 0.052 | 0.179 | 0.98 | 0.137 | 1 Teleostei | 0.79 |
| N.t82 | 558/57 | 05/07/14 | 16.6 | 18.2 | 11.4 | 27.43 | 22.37 | m | 0.08 | 0.23 | 2.987 | 0.193 | 1 Teleostei | 2.65 |
| N.t83 | 558/57 | 05/07/14 | 14.4 | 15.8 | 9.7 | 12.59 | 13.07 | m | 0.042 | 0.16 | 0.66 | 0.08 | 2 Teleostei | 0.5 |
| N.t84 | 558/57 | 05/07/14 | 18 | 19.6 | 12.1 | 32.9 | 29.3 | m | 0.19 | 0.52 | 1.106 | 0.26 | 1 Teleostei | 0.73 |
| N.t85 | 558/57 | 05/07/14 | 16.5 | 18 | 11.4 | 25.87 | 24.37 | m | 0.11 | 0.19 |  |  |  |  |
| N.t86 | 558/57 | 05/07/14 | 15 | 16.6 | 9.9 | 21.11 | 17.19 | m | 0.049 | 0.26 | 2.43 | 0.16 | 2 Teleostei | 2.19 |
| N.t87 | 558/57 | 05/07/14 | 16 | 17.3 | 10.5 | 21.7 | 19.64 | m | 0.06 | 0.22 | 0.61 | 0.19 | 2 Teleostei | 0.23 |
| N.t88 | 558/57 | 05/07/14 | 16.2 | 17.5 | 11 | 22.34 | 19.72 | f | 0.08 | 0.17 | 1.83 | 0.18 | 1 Teleostei | 1.57 |
| N.t89 | 558/57 | 05/07/14 | 15 | 16.4 | 10.3 | 17.73 | 14.92 | m | 0.054 | 0.165 | 1.45 | 0.13 | 1 Myctophidae | 1.26 |
| N.t90 | 558/57 | 05/07/14 | 17.4 | 19 | 11.9 | 29.07 | 26.1 | f | 0.23 | 0.27 | 1.05 | 0.29 | 1 Teleostei | 0.56 |
| N.t91 | 558/57 | 05/07/14 | 14.4 | 20.8 | 13.5 | 41.73 | 36.5 | f | 2.13 | 0.86 | 0.8 | 0.29 | 1 Teleostei | 0.4 |
