# Supplementary material for: Mesopredatory fishes from the subtropical upwelling region off NW-Africa characterised by their parasite fauna
Source: PeerJ. 2018 Aug 8;6:e5339. doi: 10.7717/peerj.5339 (PMC6087424; doi:10.7717/peerj.5339)
Supplement: Data S2 — Ac, Acanthocephala, A. peg, Anisakis pegreffii, A. phy, Anisakis physeteris, A. sp., Anisakis sp., A. typ, Anisakis typica; Bo, Bomolochidae; C, Cestoda; Cal , Caligidae; Cr, Crustacea; D, Digenea; Dic, Diclidophoridae, Hem, Hemiuridae, H. sp., Hysterothylacium sp.; ID, host identification code; K. thy, Kudoa thyrsites; L. mic, Lecithochirium microstomum; L. sp., Lernaeenicus sp.; M, Monogenea; My, Myxozoa; N, Nematoda; N. sp., Nybelinia sp.; O. tra, Octoplectanocotyle travassosi; Tet , Tetraphyllidea; Try, Trypanorhyncha. [file peerj-06-5339-s004.docx]

Supplemental Raw Data S2: Raw data of the parasitisation of *Trichiurus lepturus* and *Nealotus tripes* specimens. Ac = Acanthocephala, *A. peg* = *Anisakis pegreffii*, *A. phy* = *Anisakis physeteris*, *A*. sp. = *Anisakis* sp., *A. typ* = *Anisakis typica*, Bo = Bomolochidae, C = Cestoda, Cal = Caligidae, Cr = Crustacea, D = Digenea, Dic = Diclidophoridae, Hem = Hemiuridae, *H*. sp. = Hysterothylacium sp., ID= host identification code, *K. thy* = *Kudoa thyrsites*, *L. mic* = *Lecithochirium microstomum*, *L*. sp. = *Lernaeenicus* sp., M = Monogenea, My = Myxozoa, N = Nematoda, *N*. sp. = *Nybelinia* sp., *O. tra* = *Octoplectanocotyle travassosi*, Tet = Tetraphyllidea, Try = Trypanorhyncha.

| ID | My | *K. thy* | D | Hem | *L. mic* | *indet.* | M | Dic | *O. tra* | C | Tet | Try | *N*. sp. | N | *A. peg* | *A. phy* | *A. typ* | *A.* sp. | *H*. sp. | Ac | Cr | Bo | Cal | *L*. sp. |
| --- | --- | --- | --- | --- | --- | --- | --- | --- | --- | --- | --- | --- | --- | --- | --- | --- | --- | --- | --- | --- | --- | --- | --- | --- |
| T.l1 | **0** | 0 | **16** | 0 | 16 | 0 | **0** | 0 | 0 | **0** | 0 | 0 | 0 | **2** | 0 | 0 | 0 | 0 | 0 | **0** | **0** | 0 | 0 | 0 |
| T.l2 | **0** | 0 | **104** | 0 | 104 | 0 | **0** | 0 | 0 | **4** | 0 | 0 | 0 | **2** | 0 | 0 | 0 | 0 | 0 | **0** | **0** | 0 | 0 | 0 |
| T.l3 | **0** | 0 | **29** | 0 | 29 | 0 | **0** | 0 | 0 | **0** | 0 | 0 | 0 | **0** | 0 | 0 | 0 | 0 | 0 | **0** | **0** | 0 | 0 | 0 |
| T.l4 | **0** | 0 | **2** | 0 | 2 | 0 | **0** | 0 | 0 | **4** | 0 | 0 | 0 | **2** | 0 | 0 | 0 | 0 | 0 | **0** | **0** | 0 | 0 | 0 |
| T.l5 | **0** | 0 | **124** | 0 | 124 | 0 | **0** | 0 | 0 | **0** | 0 | 0 | 0 | **0** | 0 | 0 | 0 | 0 | 0 | **0** | **0** | 0 | 0 | 0 |
| T.l6 | **0** | 0 | **17** | 0 | 17 | 0 | **0** | 0 | 0 | **0** | 0 | 0 | 0 | **2** | 0 | 0 | 0 | 0 | 0 | **0** | **0** | 0 | 0 | 0 |
| T.l7 | **0** | 0 | **52** | 0 | 52 | 0 | **0** | 0 | 0 | **0** | 0 | 0 | 0 | **4** | 1 | 0 | 1 | 0 | 0 | **0** | **0** | 0 | 0 | 0 |
| T.l8 | **0** | 0 | **22** | 0 | 22 | 0 | **0** | 0 | 0 | **0** | 0 | 0 | 0 | **2** | 0 | 0 | 1 | 0 | 0 | **0** | **0** | 0 | 0 | 0 |
| T.l9 | **0** | 0 | **1** | 0 | 1 | 0 | **0** | 0 | 0 | **0** | 0 | 0 | 0 | **0** | 0 | 0 | 0 | 0 | 0 | **1** | **0** | 0 | 0 | 0 |
| T.l10 | **0** | 0 | **21** | 0 | 21 | 0 | **0** | 0 | 0 | **0** | 0 | 0 | 0 | **1** | 0 | 0 | 0 | 0 | 0 | **0** | **0** | 0 | 0 | 0 |
| T.l11 | **0** | 0 | **0** | 0 | 0 | 0 | **0** | 0 | 0 | **0** | 0 | 0 | 0 | **0** | 0 | 0 | 0 | 0 | 0 | **0** | **0** | 0 | 0 | 0 |
| T.l12 | **0** | 0 | **63** | 0 | 63 | 0 | **0** | 0 | 0 | **0** | 0 | 0 | 0 | **1** | 0 | 0 | 0 | 0 | 0 | **0** | **0** | 0 | 0 | 0 |
| T.l13 | **0** | 0 | **35** | 0 | 35 | 0 | **0** | 0 | 0 | **0** | 0 | 0 | 0 | **0** | 0 | 0 | 0 | 0 | 0 | **0** | **0** | 0 | 0 | 0 |
| T.l14 | **0** | 0 | **227** | 0 | 222 | 5 | **1** | 0 | 1 | **21** | 0 | 0 | 0 | **3** | 1 | 0 | 1 | 0 | 0 | **0** | **0** | 0 | 0 | 0 |
| T.l15 | **0** | 0 | **158** | 0 | 154 | 4 | **0** | 0 | 0 | **0** | 0 | 0 | 0 | **4** | 0 | 0 | 4 | 0 | 0 | **0** | **0** | 0 | 0 | 0 |
| T.l16 | **0** | 0 | **27** | 0 | 27 | 0 | **0** | 0 | 0 | **1** | 0 | 0 | 0 | **0** | 0 | 0 | 0 | 0 | 0 | **0** | **0** | 0 | 0 | 0 |
| T.l17 | **0** | 0 | **68** | 0 | 68 | 0 | **0** | 0 | 0 | **0** | 0 | 0 | 0 | **1** | 0 | 0 | 0 | 0 | 0 | **0** | **1** | 1 | 0 | 0 |
| T.l18 | **0** | 0 | **38** | 0 | 38 | 0 | **0** | 0 | 0 | **0** | 0 | 0 | 0 | **0** | 0 | 0 | 0 | 0 | 0 | **0** | **0** | 0 | 0 | 0 |
| T.l19 | **0** | 0 | **0** | 0 | 0 | 0 | **0** | 0 | 0 | **3** | 3 | 0 | 0 | **1** | 0 | 0 | 1 | 0 | 0 | **0** | **0** | 0 | 0 | 0 |
| T.l20 | **0** | 0 | **0** | 0 | 0 | 0 | **0** | 0 | 0 | **~2500** | 3 | 0 | 0 | **0** | 0 | 0 | 0 | 0 | 0 | **0** | **0** | 0 | 0 | 0 |
| T.l21 | **0** | 0 | **31** | 0 | 31 | 0 | **0** | 0 | 0 | **1** | 1 | 0 | 0 | **1** | 1 | 0 | 0 | 0 | 0 | **0** | **0** | 0 | 0 | 0 |
| T.l22 | **0** | 0 | **63** | 0 | 63 | 0 | **0** | 0 | 0 | **2** | 2 | 0 | 0 | **1** | 1 | 0 | 0 | 0 | 0 | **0** | **0** | 0 | 0 | 0 |
| T.l23 | **0** | 0 | **0** | 0 | 0 | 0 | **0** | 0 | 0 | **~3000** | 0 | 0 | 0 | **0** | 0 | 0 | 0 | 0 | 0 | **0** | **0** | 0 | 0 | 0 |
| T.l24 | **0** | 0 | **24** | 0 | 24 | 0 | **0** | 0 | 0 | **0** | 0 | 0 | 0 | **2** | 0 | 0 | 1 | 0 | 0 | **0** | **0** | 0 | 0 | 0 |
| T.l25 | **0** | 0 | **0** | 0 | 0 | 0 | **0** | 0 | 0 | **~6100** | 0 | 0 | 0 | **0** | 0 | 0 | 0 | 0 | 0 | **0** | **0** | 0 | 0 | 0 |
| T.l26 | **0** | 0 | **149** | 0 | 149 | 0 | **0** | 0 | 0 | **3** | 3 | 0 | 0 | **0** | 0 | 0 | 0 | 0 | 0 | **0** | **0** | 0 | 0 | 0 |
| T.l27 | **0** | 0 | **39** | 0 | 38 | 1 | **2** | 0 | 2 | **0** | 0 | 0 | 0 | **0** | 0 | 0 | 0 | 0 | 0 | **0** | **0** | 0 | 0 | 0 |
| T.l28 | **0** | 0 | **47** | 0 | 47 | 0 | **0** | 0 | 0 | **~1000** | 1 | 0 | 0 | **1** | 1 | 0 | 0 | 0 | 0 | **0** | **0** | 0 | 0 | 0 |
| T.l29 | **0** | 0 | **0** | 0 | 0 | 0 | **0** | 0 | 0 | **~3000** | 18 | 0 | 0 | **1** | 0 | 0 | 0 | 0 | 0 | **0** | **0** | 0 | 0 | 0 |
| T.l30 | **0** | 0 | **74** | 0 | 74 | 0 | **6** | 0 | 6 | **0** | 0 | 0 | 0 | **0** | 0 | 0 | 0 | 0 | 0 | **0** | **0** | 0 | 0 | 0 |
| T.l31 | **0** | 0 | **18** | 0 | 18 | 0 | **0** | 0 | 0 | **0** | 0 | 0 | 0 | **1** | 0 | 0 | 0 | 0 | 0 | **0** | **0** | 0 | 0 | 0 |
| T.l32 | **0** | 0 | **121** | 0 | 121 | 0 | **2** | 0 | 2 | **0** | 0 | 0 | 0 | **3** | 3 | 0 | 0 | 0 | 0 | **0** | **0** | 0 | 0 | 0 |
| T.l33 | **0** | 0 | **73** | 0 | 73 | 0 | **0** | 0 | 0 | **0** | 0 | 0 | 0 | **4** | 1 | 0 | 3 | 0 | 0 | **0** | **1** | 0 | 1 | 0 |
| T.l34 | **0** | 0 | **10** | 0 | 10 | 0 | **1** | 0 | 1 | **149** | 149 | 0 | 0 | **0** | 0 | 0 | 0 | 0 | 0 | **0** | **0** | 0 | 0 | 0 |
| T.l35 | **0** | 0 | **50** | 0 | 50 | 0 | **0** | 0 | 0 | **0** | 0 | 0 | 0 | **0** | 0 | 0 | 0 | 0 | 0 | **0** | **0** | 0 | 0 | 0 |
| T.l36 | **0** | 0 | **77** | 0 | 77 | 0 | **0** | 0 | 0 | **0** | 0 | 0 | 0 | **1** | 0 | 0 | 1 | 0 | 0 | **0** | **0** | 0 | 0 | 0 |
| T.l37 | **0** | 0 | **1** | 0 | 1 | 0 | **0** | 0 | 0 | **2** | 2 | 0 | 0 | **0** | 0 | 0 | 0 | 0 | 0 | **0** | **0** | 0 | 0 | 0 |
| T.l38 | **0** | 0 | **40** | 0 | 40 | 0 | **0** | 0 | 0 | **0** | 0 | 0 | 0 | **1** | 1 | 0 | 0 | 0 | 0 | **0** | **0** | 0 | 0 | 0 |
| T.l39 | **0** | 0 | **1** | 0 | 1 | 0 | **0** | 0 | 0 | **~2500** | 0 | 0 | 0 | **2** | 0 | 0 | 0 | 0 | 0 | **0** | **0** | 0 | 0 | 0 |
| T.l40 | **0** | 0 | **91** | 0 | 91 | 0 | **0** | 0 | 0 | **4** | 4 | 0 | 0 | **0** | 0 | 0 | 0 | 0 | 0 | **0** | **1** | 0 | 1 | 0 |
| T.l41 | **0** | 0 | **1** | 0 | 1 | 0 | **0** | 0 | 0 | **~3500** | 0 | 0 | 0 | **4** | 0 | 0 | 0 | 0 | 2 | **0** | **0** | 0 | 0 | 0 |
| T.l42 | **0** | 0 | **0** | 0 | 0 | 0 | **0** | 0 | 0 | **~1000** | 0 | 0 | 0 | **0** | 0 | 0 | 0 | 0 | 0 | **0** | **1** | 0 | 1 | 0 |
| T.l43 | **0** | 0 | **39** | 0 | 39 | 0 | **0** | 0 | 0 | **1** | 1 | 0 | 0 | **1** | 0 | 0 | 1 | 0 | 0 | **0** | **0** | 0 | 0 | 0 |
| T.l44 | **0** | 0 | **105** | 0 | 104 | 1 | **0** | 0 | 0 | **0** | 0 | 0 | 0 | **0** | 0 | 0 | 0 | 0 | 0 | **0** | **0** | 0 | 0 | 0 |
| T.l45 | **0** | 0 | **29** | 0 | 29 | 0 | **0** | 0 | 0 | **0** | 0 | 0 | 0 | **0** | 0 | 0 | 0 | 0 | 0 | **0** | **0** | 0 | 0 | 0 |
| T.l46 | **0** | 0 | **45** | 0 | 45 | 0 | **0** | 0 | 0 | **6** | 0 | 5 | 0 | **4** | 1 | 0 | 2 | 0 | 0 | **0** | **0** | 0 | 0 | 0 |
| T.l47 | **0** | 0 | **88** | 0 | 88 | 0 | **1** | 0 | 1 | **0** | 0 | 0 | 0 | **0** | 0 | 0 | 0 | 0 | 0 | **0** | **1** | 0 | 1 | 0 |
| T.l48 | **0** | 0 | **148** | 0 | 148 | 0 | **1** | 0 | 1 | **1** | 0 | 0 | 0 | **0** | 0 | 0 | 0 | 0 | 0 | **0** | **2** | 0 | 2 | 0 |
| T.l49 | **0** | 0 | **138** | 0 | 138 | 0 | **0** | 0 | 0 | **0** | 0 | 0 | 0 | **0** | 0 | 0 | 0 | 0 | 0 | **0** | **0** | 0 | 0 | 0 |
| T.l50 | **0** | 0 | **1** | 0 | 1 | 0 | **0** | 0 | 0 | **~1900** | 362 | 0 | 0 | **1** | 0 | 0 | 0 | 0 | 1 | **0** | **0** | 0 | 0 | 0 |
| T.l51 | **0** | 0 | **0** | 0 | 0 | 0 | **0** | 0 | 0 | **~1800** | 1 | 0 | 0 | **1** | 0 | 0 | 0 | 0 | 0 | **0** | **0** | 0 | 0 | 0 |
| T.l52 | **0** | 0 | **81** | 0 | 81 | 0 | **0** | 0 | 0 | **1** | 0 | 0 | 0 | **6** | 1 | 0 | 1 | 0 | 0 | **0** | **0** | 0 | 0 | 0 |
| T.l53 | **0** | 0 | **0** | 0 | 0 | 0 | **0** | 0 | 0 | **0** | 0 | 0 | 0 | **2** | 0 | 0 | 1 | 0 | 0 | **0** | **0** | 0 | 0 | 0 |
| T.l54 | **0** | 0 | **100** | 0 | 100 | 0 | **1** | 0 | 1 | **7** | 6 | 0 | 0 | **1** | 0 | 0 | 0 | 0 | 0 | **0** | **0** | 0 | 0 | 0 |
| T.l55 | **0** | 0 | **85** | 0 | 85 | 0 | **0** | 0 | 0 | **20** | 20 | 0 | 0 | **1** | 1 | 0 | 0 | 0 | 0 | **0** | **0** | 0 | 0 | 0 |
| T.l56 | **0** | 0 | **57** | 0 | 56 | 1 | **0** | 0 | 0 | **0** | 0 | 0 | 0 | **1** | 0 | 0 | 1 | 0 | 0 | **0** | **0** | 0 | 0 | 0 |
| T.l57 | **0** | 0 | **1** | 0 | 1 | 0 | **0** | 0 | 0 | **~4300** | 2 | 0 | 0 | **0** | 0 | 0 | 0 | 0 | 0 | **0** | **0** | 0 | 0 | 0 |
| T.l58 | **0** | 0 | **85** | 0 | 85 | 0 | **1** | 0 | 1 | **1** | 0 | 0 | 0 | **2** | 1 | 0 | 1 | 0 | 0 | **0** | **0** | 0 | 0 | 0 |
| T.l59 | **0** | 0 | **94** | 0 | 94 | 0 | **0** | 0 | 0 | **0** | 0 | 0 | 0 | **2** | 2 | 0 | 0 | 0 | 0 | **0** | **0** | 0 | 0 | 0 |
| T.l60 | **0** | 0 | **0** | 0 | 0 | 0 | **0** | 0 | 0 | **~5800** | 0 | 0 | 0 | **2** | 0 | 0 | 0 | 0 | 0 | **0** | **0** | 0 | 0 | 0 |
| T.l61 | **0** | 0 | **30** | 0 | 30 | 0 | **1** | 0 | 1 | **5** | 5 | 0 | 0 | **0** | 0 | 0 | 0 | 0 | 0 | **0** | **0** | 0 | 0 | 0 |
| T.l62 | **0** | 0 | **45** | 0 | 45 | 0 | **0** | 0 | 0 | **2** | 2 | 0 | 0 | **14** | 0 | 0 | 0 | 0 | 0 | **0** | **1** | 0 | 1 | 0 |
| T.l63 | **0** | 0 | **1** | 0 | 1 | 0 | **0** | 0 | 0 | **~5300** | 1 | 0 | 0 | **2** | 0 | 0 | 0 | 0 | 0 | **0** | **0** | 0 | 0 | 0 |
| T.l64 | **0** | 0 | **1** | 0 | 1 | 0 | **0** | 0 | 0 | **~3900** | 5 | 0 | 0 | **0** | 0 | 0 | 0 | 0 | 0 | **0** | **0** | 0 | 0 | 0 |
| T.l65 | **0** | 0 | **125** | 0 | 125 | 0 | **1** | 0 | 1 | **404** | 4 | 0 | 0 | **3** | 1 | 0 | 2 | 0 | 0 | **0** | **0** | 0 | 0 | 0 |
| T.l66 | **0** | 0 | **2** | 0 | 1 | 1 | **0** | 0 | 0 | **519** | 5 | 0 | 0 | **0** | 0 | 0 | 0 | 0 | 0 | **0** | **0** | 0 | 0 | 0 |
| T.l67 | **0** | 0 | **56** | 0 | 56 | 0 | **0** | 0 | 0 | **2** | 2 | 0 | 0 | **0** | 0 | 0 | 0 | 0 | 0 | **0** | **0** | 0 | 0 | 0 |
| T.l68 | **0** | 0 | **48** | 0 | 47 | 1 | **1** |  | 1 | **3** | 0 | 0 | 0 | **2** | 0 | 0 | 2 | 0 | 0 | **0** | **0** | 0 | 0 | 0 |
| T.l69 | **0** | 0 | **168** | 0 | 168 | 0 | **1** | 0 | 1 | **5** | 5 | 0 | 0 | **0** | 0 | 0 | 0 | 0 | 0 | **0** | **0** | 0 | 0 | 0 |
| T.l70 | **0** | 0 | **60** | 0 | 60 | 0 | **0** | 0 | 0 | **3** | 0 | 0 | 0 | **1** | 1 | 0 | 0 | 0 | 0 | **0** | **0** | 0 | 0 | 0 |
| T.l71 | **0** | 0 | **0** | 0 | 0 | 0 | **0** | 0 | 0 | **9** | 9 | 0 | 0 | **0** | 0 | 0 | 0 | 0 | 0 | **1** | **0** | 0 | 0 | 0 |
| T.l72 | **0** | 0 | **33** | 0 | 33 | 0 | **0** | 0 | 0 | **0** | 0 | 0 | 0 | **1** | 0 | 0 | 0 | 0 | 0 | **0** | **0** | 0 | 0 | 0 |
| T.l73 | **0** | 0 | **1** | 0 | 1 | 0 | **0** | 0 | 0 | **0** | 0 | 0 | 0 | **0** | 0 | 0 | 0 | 0 | 0 | **0** | **0** | 0 | 0 | 0 |
| T.l74 | **0** | 0 | **53** | 0 | 53 | 0 | **0** | 0 | 0 | **9** | 9 | 0 | 0 | **1** | 1 | 0 | 0 | 0 | 0 | **0** | **0** | 0 | 0 | 0 |
| T.l75 | **0** | 0 | **47** | 0 | 47 | 0 | **0** | 0 | 0 | **0** | 0 | 0 | 0 | **0** | 0 | 0 | 0 | 0 | 0 | **0** | **0** | 0 | 0 | 0 |
| T.l76 | **0** | 0 | **64** | 0 | 64 | 0 | **0** | 0 | 0 | **0** | 0 | 0 | 0 | **0** | 0 | 0 | 0 | 0 | 0 | **0** | **0** | 0 | 0 | 0 |
| T.l77 | **0** | 0 | **98** | 0 | 98 | 0 | **0** | 0 | 0 | **0** | 0 | 0 | 0 | **2** | 0 | 0 | 0 | 0 | 0 | **0** | **0** | 0 | 0 | 0 |
| T.l78 | **0** | 0 | **145** | 0 | 145 | 0 | **0** | 0 | 0 | **0** | 0 | 0 | 0 | **1** | 1 | 0 | 0 | 0 | 0 | **0** | **0** | 0 | 0 | 0 |
| T.l79 | **0** | 0 | **64** | 0 | 64 | 0 | **0** | 0 | 0 | **97** | 28 | 0 | 0 | **1** | 1 | 0 | 0 | 0 | 0 | **0** | **0** | 0 | 0 | 0 |
| T.l80 | **0** | 0 | **77** | 0 | 77 | 0 | **0** | 0 | 0 | **0** | 0 | 0 | 0 | **0** | 0 | 0 | 0 | 0 | 0 | **0** | **0** | 0 | 0 | 0 |
| T.l81 | **0** | 0 | **3** | 0 | 3 | 0 | **0** | 0 | 0 | **0** | 0 | 0 | 0 | **4** | 0 | 0 | 0 | 0 | 0 | **0** | **0** | 0 | 0 | 0 |
| T.l82 | **0** | 0 | **42** | 0 | 42 | 0 | **0** | 0 | 0 | **9** | 9 | 0 | 0 | **0** | 0 | 0 | 0 | 0 | 0 | **0** | **0** | 0 | 0 | 0 |
| T.l83 | **0** | 0 | **52** | 0 | 52 | 0 | **0** | 0 | 0 | **3** | 3 | 0 | 0 | **0** | 0 | 0 | 0 | 0 | 0 | **0** | **0** | 0 | 0 | 0 |
| T.l84 | **0** | 0 | **34** | 0 | 34 | 0 | **0** | 0 | 0 | **0** | 0 | 0 | 0 | **0** | 0 | 0 | 0 | 0 | 0 | **0** | **0** | 0 | 0 | 0 |
| T.l85 | **0** | 0 | **33** | 0 | 33 | 0 | **0** | 0 | 0 | **0** | 0 | 0 | 0 | **2** | 1 | 0 | 0 | 0 | 1 | **0** | **0** | 0 | 0 | 0 |
| T.l86 | **0** | 0 | **16** | 0 | 16 | 0 | **0** | 0 | 0 | **0** | 0 | 0 | 0 | **0** | 0 | 0 | 0 | 0 | 0 | **0** | **0** | 0 | 0 | 0 |
| T.l87 | **0** | 0 | **35** | 0 | 35 | 0 | **0** | 0 | 0 | **11** | 5 | 0 | 0 | **0** | 0 | 0 | 0 | 0 | 0 | **0** | **0** | 0 | 0 | 0 |
| T.l88 | **0** | 0 | **114** | 0 | 114 | 0 | **0** | 0 | 0 | **0** | 0 | 0 | 0 | **0** | 0 | 0 | 0 | 0 | 0 | **0** | **0** | 0 | 0 | 0 |
| T.l89 | **0** | 0 | **17** | 0 | 17 | 0 | **0** | 0 | 0 | **4** | 3 | 0 | 0 | **0** | 0 | 0 | 0 | 0 | 0 | **0** | **0** | 0 | 0 | 0 |
| T.l90 | **0** | 0 | **0** | 0 | 0 | 0 | **0** | 0 | 0 | **~1400** | 0 | 0 | 0 | **0** | 0 | 0 | 0 | 0 | 0 | **0** | **0** | 0 | 0 | 0 |
| T.l91 | **0** | 0 | **17** | 0 | 17 | 0 | **0** | 0 | 0 | **0** | 0 | 0 | 0 | **2** | 1 | 0 | 0 | 0 | 0 | **0** | **0** | 0 | 0 | 0 |
| T.l92 | **0** | 0 | **111** | 0 | 111 | 0 | **0** | 0 | 0 | **172** | 172 | 0 | 0 | **2** | 2 | 0 | 0 | 0 | 0 | **0** | **0** | 0 | 0 | 0 |
| T.l93 | **0** | 0 | **69** | 0 | 69 | 0 | **0** | 0 | 0 | **309** | 4 | 0 | 0 | **2** | 0 | 0 | 2 | 0 | 0 | **0** | **0** | 0 | 0 | 0 |
| T.l94 | **0** | 0 | **0** | 0 | 0 | 0 | **0** | 0 | 0 | **~700** | 0 | 0 | 0 | **1** | 1 | 0 | 0 | 0 | 0 | **0** | **0** | 0 | 0 | 0 |
| T.l95 | **0** | 0 | **77** | 1 | 76 | 0 | **0** | 0 | 0 | **0** | 0 | 0 | 0 | **0** | 0 | 0 | 0 | 0 | 0 | **0** | **0** | 0 | 0 | 0 |
| T.l96 | **0** | 0 | **106** | 0 | 106 | 0 | **0** | 0 | 0 | **~900** | 1 | 0 | 0 | **0** | 0 | 0 | 0 | 0 | 0 | **0** | **0** | 0 | 0 | 0 |
| T.l97 | **0** | 0 | **104** | 0 | 104 | 0 | **0** | 0 | 0 | **23** | 23 | 0 | 0 | **0** | 0 | 0 | 0 | 0 | 0 | **0** | **0** | 0 | 0 | 0 |
| T.l98 | **0** | 0 | **87** | 0 | 87 | 0 | **0** | 0 | 0 | **57** | 2 | 0 | 0 | **1** | 1 | 0 | 0 | 0 | 0 | **0** | **0** | 0 | 0 | 0 |
| T.l99 | **0** | 0 | **124** | 0 | 124 | 0 | **0** | 0 | 0 | **0** | 0 | 0 | 0 | **0** | 0 | 0 | 0 | 0 | 0 | **0** | **0** | 0 | 0 | 0 |
| T.l100 | **0** | 0 | **69** | 0 | 69 | 0 | **0** | 0 | 0 | **0** | 0 | 0 | 0 | **1** | 0 | 0 | 1 | 0 | 0 | **0** | **0** | 0 | 0 | 0 |
| T.l101 | **0** | 0 | **47** | 0 | 47 | 0 | **0** | 0 | 0 | **3** | 3 | 0 | 0 | **0** | 0 | 0 | 0 | 0 | 0 | **0** | **0** | 0 | 0 | 0 |
| T.l102 | **0** | 0 | **45** | 0 | 45 | 0 | **0** | 0 | 0 | **0** | 0 | 0 | 0 | **0** | 0 | 0 | 0 | 0 | 0 | **0** | **0** | 0 | 0 | 0 |
| T.l103 | **0** | 0 | **52** | 0 | 52 | 0 | **0** | 0 | 0 | **0** | 0 | 0 | 0 | **1** | 1 | 0 | 0 | 0 | 0 | **0** | **0** | 0 | 0 | 0 |
| T.l104 | **0** | 0 | **28** | 0 | 28 | 0 | **0** | 0 | 0 | **0** | 0 | 0 | 0 | **0** | 0 | 0 | 0 | 0 | 0 | **0** | **0** | 0 | 0 | 0 |
| N.t1 | **0** | 0 | **0** | 0 | 0 | 0 | **0** | 0 | 0 | **~13500** | 216 | 0 | 0 | **0** | 0 | 0 | 0 | 0 | 0 | **0** | **0** | 0 | 0 | 0 |
| N.t2 | ***** | * | **0** | 0 | 0 | 0 | **3** | 3 | 0 | **106** | 0 | 0 | 0 | **0** | 0 | 0 | 0 | 0 | 0 | **0** | **0** | 0 | 0 | 0 |
| N.t3 | **0** | 0 | **0** | 0 | 0 | 0 | **2** | 2 | 0 | **0** | 0 | 0 | 0 | **0** | 0 | 0 | 0 | 0 | 0 | **0** | **0** | 0 | 0 | 0 |
| N.t4 | **0** | 0 | **1** | 0 | 0 | 1 | **1** | 1 | 0 | **4** | 1 | 0 | 0 | **1** | 0 | 0 | 0 | 0 | 0 | **0** | **0** | 0 | 0 | 0 |
| N.t5 | **0** | 0 | **0** | 0 | 0 | 0 | **1** | 1 | 0 | **28** | 27 | 0 | 1 | **0** | 0 | 0 | 0 | 0 | 0 | **0** | **0** | 0 | 0 | 0 |
| N.t6 | **0** | 0 | **0** | 0 | 0 | 0 | **0** | 0 | 0 | **0** | 0 | 0 | 0 | **0** | 0 | 0 | 0 | 0 | 0 | **0** | **0** | 0 | 0 | 0 |
| N.t7 | **0** | 0 | **0** | 0 | 0 | 0 | **0** | 0 | 0 | **2** | 2 | 0 | 0 | **0** | 0 | 0 | 0 | 0 | 0 | **0** | **0** | 0 | 0 | 0 |
| N.t8 | **0** | 0 | **1** | 0 | 0 | 1 | **0** | 0 | 0 | **0** | 0 | 0 | 0 | **0** | 0 | 0 | 0 | 0 | 0 | **0** | **0** | 0 | 0 | 0 |
| N.t9 | **0** | 0 | **1** | 0 | 0 | 1 | **0** | 0 | 0 | **92** | 35 | 0 | 0 | **0** | 0 | 0 | 0 | 0 | 0 | **0** | **0** | 0 | 0 | 0 |
| N.t10 | **0** | 0 | **0** | 0 | 0 | 0 | **0** | 0 | 0 | **1** | 1 | 0 | 0 | **0** | 0 | 0 | 0 | 0 | 0 | **1** | **0** | 0 | 0 | 0 |
| N.t11 | **0** | 0 | **0** | 0 | 0 | 0 | **1** | 1 | 0 | **27** | 0 | 0 | 0 | **1** | 0 | 0 | 0 | 0 | 0 | **0** | **1** | 1 | 0 | 0 |
| N.t12 | **0** | 0 | **4** | 0 | 0 | 4 | **1** | 1 | 0 | **3** | 1 | 0 | 0 | **4** | 0 | 0 | 1 | 1 | 0 | **0** | **0** | 0 | 0 | 0 |
| N.t13 | **0** | 0 | **3** | 0 | 0 | 3 | **0** | 0 | 0 | **79** | 79 | 0 | 0 | **0** | 0 | 0 | 0 | 0 | 0 | **0** | **0** | 0 | 0 | 0 |
| N.t14 | **0** | 0 | **0** | 0 | 0 | 0 | **1** | 1 | 0 | **8** | 8 | 0 | 0 | **0** | 0 | 0 | 0 | 0 | 0 | **0** | **0** | 0 | 0 | 0 |
| N.t15 | **0** | 0 | **1** | 0 | 0 | 1 | **5** | 5 | 0 | **22** | 22 | 0 | 0 | **2** | 0 | 0 | 0 | 2 | 0 | **0** | **0** | 0 | 0 | 0 |
| N.t16 | **0** | 0 | **0** | 0 | 0 | 0 | **0** | 0 | 0 | **2** | 2 | 0 | 0 | **0** | 0 | 0 | 0 | 0 | 0 | **0** | **0** | 0 | 0 | 0 |
| N.t17 | **0** | 0 | **0** | 0 | 0 | 0 | **3** | 3 | 0 | **~500** | 0 | 0 | 0 | **3** | 0 | 0 | 0 | 0 | 0 | **0** | **0** | 0 | 0 | 0 |
| N.t18 | **0** | 0 | **105** | 0 | 0 | 105 | **2** | 2 | 0 | **0** | 0 | 0 | 2 | **0** | 0 | 0 | 0 | 0 | 0 | **0** | **0** | 0 | 0 | 0 |
| N.t19 | **0** | 0 | **2** | 0 | 0 | 2 | **3** | 3 | 0 | **156** | 156 | 0 | 0 | **4** | 0 | 1 | 0 | 0 | 0 | **0** | **0** | 0 | 0 | 0 |
| N.t20 | **0** | 0 | **26** | 0 | 0 | 26 | **0** | 0 | 0 | **86** | 86 | 0 | 0 | **0** | 0 | 0 | 0 | 0 | 0 | **0** | **0** | 0 | 0 | 0 |
| N.t21 | **0** | 0 | **0** | 0 | 0 | 0 | **0** | 0 | 0 | **48** | 48 | 0 | 0 | **0** | 0 | 0 | 0 | 0 | 0 | **0** | **0** | 0 | 0 | 0 |
| N.t22 | **0** | 0 | **20** | 0 | 0 | 20 | **1** | 1 | 0 | **19** | 19 | 0 | 0 | **0** | 0 | 0 | 0 | 0 | 0 | **0** | **0** | 0 | 0 | 0 |
| N.t23 | **0** | 0 | **27** | 0 | 0 | 27 | **1** | 1 | 0 | **31** | 31 | 0 | 0 | **0** | 0 | 0 | 0 | 0 | 0 | **0** | **0** | 0 | 0 | 0 |
| N.t24 | **0** | 0 | **10** | 0 | 0 | 10 | **1** | 1 | 0 | **246** | 246 | 0 | 0 | **1** | 0 | 1 | 0 | 0 | 0 | **0** | **0** | 0 | 0 | 0 |
| N.t25 | **0** | 0 | **3** | 0 | 0 | 3 | **2** | 2 | 0 | **2** | 2 | 0 | 0 | **0** | 0 | 0 | 0 | 0 | 0 | **0** | **0** | 0 | 0 | 0 |
| N.t26 | **0** | 0 | **1** | 0 | 0 | 1 | **0** | 0 | 0 | **0** | 0 | 0 | 0 | **0** | 0 | 0 | 0 | 0 | 0 | **0** | **0** | 0 | 0 | 0 |
| N.t27 | **0** | 0 | **1** | 0 | 0 | 1 | **0** | 0 | 0 | **11** | 8 | 0 | 0 | **0** | 0 | 0 | 0 | 0 | 0 | **0** | **2** | 2 | 0 | 0 |
| N.t28 | **0** | 0 | **0** | 0 | 0 | 0 | **0** | 0 | 0 | **7** | 0 | 0 | 0 | **4** | 0 | 0 | 2 | 0 | 0 | **0** | **1** | 1 | 0 | 0 |
| N.t29 | **0** | 0 | **0** | 0 | 0 | 0 | **1** | 1 | 0 | **28** | 27 | 0 | 1 | **0** | 0 | 0 | 0 | 0 | 0 | **0** | **0** | 0 | 0 | 0 |
| N.t30 | **0** | 0 | **0** | 0 | 0 | 0 | **0** | 0 | 0 | **0** | 0 | 0 | 0 | **0** | 0 | 0 | 0 | 0 | 0 | **0** | **0** | 0 | 0 | 0 |
| N.t31 | **0** | 0 | **11** | 0 | 0 | 11 | **0** | 0 | 0 | **12** | 12 | 0 | 0 | **0** | 0 | 0 | 0 | 0 | 0 | **0** | **0** | 0 | 0 | 0 |
| N.t32 | **0** | 0 | **1** | 0 | 0 | 1 | **7** | 7 | 0 | **~1100** | 2 | 0 | 0 | **1** | 0 | 0 | 0 | 1 | 0 | **0** | **2** | 2 | 0 | 0 |
| N.t33 | **0** | 0 | **18** | 0 | 0 | 18 | **2** | 2 | 0 | **392** | 5 | 0 | 0 | **1** | 0 | 0 | 1 | 0 | 0 | **0** | **0** | 0 | 0 | 0 |
| N.t34 | **0** | 0 | **1** | 1 | 0 | 0 | **1** | 1 | 0 | **966** | 2 | 0 | 0 | **2** | 0 | 0 | 0 | 0 | 0 | **0** | **1** | 1 | 0 | 0 |
| N.t35 | **0** | 0 | **0** | 0 | 0 | 0 | **0** | 0 | 0 | **0** | 0 | 0 | 0 | **0** | 0 | 0 | 0 | 0 | 0 | **0** | **0** | 0 | 0 | 0 |
| N.t36 | **0** | 0 | **0** | 0 | 0 | 0 | **2** | 2 | 0 | **3** | 3 | 0 | 0 | **2** | 0 | 0 | 1 | 0 | 1 | **0** | **0** | 0 | 0 | 0 |
| N.t37 | **0** | 0 | **0** | 0 | 0 | 0 | **0** | 0 | 0 | **0** | 0 | 0 | 0 | **1** | 0 | 0 | 1 | 0 | 0 | **0** | **0** | 0 | 0 | 0 |
| N.t38 | **0** | 0 | **0** | 0 | 0 | 0 | **2** | 2 | 0 | **0** | 0 | 0 | 0 | **2** | 0 | 0 | 1 | 1 | 0 | **0** | **0** | 0 | 0 | 0 |
| N.t39 | **0** | 0 | **1** | 0 | 0 | 1 | **1** | 1 | 0 | **371** | 0 | 0 | 0 | **0** | 0 | 0 | 0 | 0 | 0 | **0** | **2** | 2 | 0 | 0 |
| N.t40 | **0** | 0 | **0** | 0 | 0 | 0 | **1** | 1 | 0 | **0** | 0 | 0 | 0 | **1** | 0 | 0 | 1 | 0 | 0 | **0** | **1** | 1 | 0 | 0 |
| N.t41 | **0** | 0 | **100** | 0 | 0 | 100 | **0** | 0 | 0 | **83** | 0 | 0 | 0 | **0** | 0 | 0 | 0 | 0 | 0 | **0** | **0** | 0 | 0 | 0 |
| N.t42 | **0** | 0 | **27** | 0 | 0 | 27 | **0** | 0 | 0 | **0** | 0 | 0 | 0 | **0** | 0 | 0 | 0 | 0 | 0 | **0** | **0** | 0 | 0 | 0 |
| N.t43 | **0** | 0 | **1** | 0 | 0 | 1 | **0** | 0 | 0 | **22** | 10 | 0 | 0 | **36** | 0 | 0 | 0 | 0 | 0 | **1** | **0** | 0 | 0 | 0 |
| N.t44 | **0** | 0 | **0** | 0 | 0 | 0 | **0** | 0 | 0 | **78** | 59 | 0 | 1 | **0** | 0 | 0 | 0 | 0 | 0 | **1** | **1** | 1 | 0 | 0 |
| N.t45 | **0** | 0 | **20** | 0 | 0 | 20 | **2** | 2 | 0 | **33** | 33 | 0 | 0 | **0** | 0 | 0 | 0 | 0 | 0 | **0** | **2** | 2 | 0 | 0 |
| N.t46 | **0** | 0 | **18** | 0 | 0 | 18 | **1** | 1 | 0 | **282** | 3 | 0 | 0 | **10** | 0 | 0 | 0 | 0 | 0 | **0** | **0** | 0 | 0 | 0 |
| N.t47 | **0** | 0 | **6** | 0 | 0 | 6 | **0** | 0 | 0 | **7** | 7 | 0 | 0 | **0** | 0 | 0 | 0 | 0 | 0 | **0** | **0** | 0 | 0 | 0 |
| N.t48 | **0** | 0 | **6** | 0 | 0 | 6 | **0** | 0 | 0 | **47** | 1 | 0 | 0 | **1** | 0 | 0 | 0 | 0 | 0 | **0** | **1** | 1 | 0 | 0 |
| N.t49 | **0** | 0 | **0** | 0 | 0 | 0 | **0** | 0 | 0 | **18** | 0 | 0 | 0 | **0** | 0 | 0 | 0 | 0 | 0 | **1** | **0** | 0 | 0 | 0 |
| N.t50 | **0** | 0 | **41** | 0 | 0 | 41 | **2** | 2 | 0 | **0** | 0 | 0 | 0 | **0** | 0 | 0 | 0 | 0 | 0 | **0** | **0** | 0 | 0 | 0 |
| N.t51 | **0** | 0 | **33** | 0 | 0 | 33 | **2** | 2 | 0 | **29** | 0 | 0 | 1 | **0** | 0 | 0 | 0 | 0 | 0 | **0** | **0** | 0 | 0 | 0 |
| N.t52 | **0** | 0 | **0** | 0 | 0 | 0 | **0** | 0 | 0 | **0** | 0 | 0 | 0 | **0** | 0 | 0 | 0 | 0 | 0 | **0** | **2** | 2 | 0 | 0 |
| N.t53 | **0** | 0 | **0** | 0 | 0 | 0 | **0** | 0 | 0 | **0** | 0 | 0 | 0 | **0** | 0 | 0 | 0 | 0 | 0 | **0** | **1** | 1 | 0 | 0 |
| N.t54 | **0** | 0 | **0** | 0 | 0 | 0 | **0** | 0 | 0 | **95** | 0 | 0 | 0 | **0** | 0 | 0 | 0 | 0 | 0 | **0** | **1** | 1 | 0 | 0 |
| N.t55 | **0** | 0 | **1** | 0 | 0 | 1 | **1** | 1 | 0 | **25** | 0 | 0 | 0 | **0** | 0 | 0 | 0 | 0 | 0 | **0** | **0** | 0 | 0 | 0 |
| N.t56 | **0** | 0 | **0** | 0 | 0 | 0 | **0** | 0 | 0 | **11** | 11 | 0 | 0 | **2** | 0 | 0 | 1 | 0 | 0 | **0** | **0** | 0 | 0 | 0 |
| N.t57 | **0** | 0 | **6** | 0 | 0 | 6 | **1** | 1 | 0 | **32** | 32 | 0 | 0 | **0** | 0 | 0 | 0 | 0 | 0 | **0** | **1** | 1 | 0 | 0 |
| N.t58 | **0** | 0 | **23** | 0 | 0 | 23 | **1** | 1 | 0 | **116** | 116 | 0 | 0 | **0** | 0 | 0 | 0 | 0 | 0 | **0** | **0** | 0 | 0 | 0 |
| N.t59 | **0** | 0 | **0** | 0 | 0 | 0 | **0** | 0 | 0 | **2** | 0 | 0 | 1 | **0** | 0 | 0 | 0 | 0 | 0 | **1** | **0** | 0 | 0 | 0 |
| N.t60 | **0** | 0 | **2** | 0 | 0 | 2 | **1** | 1 | 0 | **1** | 0 | 1 | 0 | **1** | 0 | 0 | 0 | 0 | 0 | **0** | **0** | 0 | 0 | 0 |
| N.t61 | **0** | 0 | **0** | 0 | 0 | 0 | **1** | 1 | 0 | **1** | 0 | 1 | 0 | **0** | 0 | 0 | 0 | 0 | 0 | **0** | **1** | 1 | 0 | 0 |
| N.t62 | **0** | 0 | **10** | 0 | 0 | 10 | **1** | 1 | 0 | **3** | 3 | 0 | 0 | **0** | 0 | 0 | 0 | 0 | 0 | **0** | **0** | 0 | 0 | 0 |
| N.t63 | **0** | 0 | **0** | 0 | 0 | 0 | **0** | 0 | 0 | **1** | 1 | 0 | 0 | **1** | 0 | 0 | 1 | 0 | 0 | **0** | **0** | 0 | 0 | 0 |
| N.t64 | **0** | 0 | **0** | 0 | 0 | 0 | **1** | 1 | 0 | **6** | 6 | 0 | 0 | **1** | 0 | 0 | 1 | 0 | 0 | **0** | **0** | 0 | 0 | 0 |
| N.t65 | **0** | 0 | **8** | 1 | 0 | 7 | **0** | 0 | 0 | **6** | 6 | 0 | 0 | **2** | 0 | 2 | 0 | 0 | 0 | **0** | **1** | 1 | 0 | 0 |
| N.t66 | **0** | 0 | **0** | 0 | 0 | 0 | **1** | 1 | 0 | **71** | 0 | 0 | 0 | **1** | 0 | 0 | 1 | 0 | 0 | **0** | **0** | 0 | 0 | 0 |
| N.t67 | **0** | 0 | **3** | 0 | 0 | 3 | **1** | 1 | 0 | **4** | 0 | 0 | 0 | **1** | 0 | 0 | 0 | 0 | 0 | **0** | **2** | 2 | 0 | 0 |
| N.t68 | **0** | 0 | **2** | 0 | 0 | 2 | **1** | 1 | 0 | **0** | 0 | 0 | 0 | **0** | 0 | 0 | 0 | 0 | 0 | **0** | **0** | 0 | 0 | 0 |
| N.t69 | **0** | 0 | **0** | 0 | 0 | 0 | **1** | 1 | 0 | **1** | 0 | 0 | 0 | **0** | 0 | 0 | 0 | 0 | 0 | **0** | **0** | 0 | 0 | 0 |
| N.t70 | **0** | 0 | **0** | 0 | 0 | 0 | **1** | 1 | 0 | **28** | 18 | 0 | 0 | **1** | 0 | 0 | 1 | 0 | 0 | **0** | **1** | 1 | 0 | 0 |
| N.t71 | **0** | 0 | **0** | 0 | 0 | 0 | **2** | 2 | 0 | **19** | 11 | 0 | 0 | **0** | 0 | 0 | 0 | 0 | 0 | **0** | **1** | 1 | 0 | 0 |
| N.t72 | **0** | 0 | **1** | 0 | 0 | 1 | **0** | 0 | 0 | **4** | 1 | 0 | 0 | **1** | 0 | 0 | 1 | 0 | 0 | **0** | **1** | 1 | 0 | 0 |
| N.t73 | **0** | 0 | **1** | 0 | 0 | 1 | **2** | 2 | 0 | **5** | 4 | 0 | 0 | **2** | 0 | 0 | 0 | 0 | 0 | **0** | **0** | 0 | 0 | 0 |
| N.t74 | **0** | 0 | **1** | 0 | 0 | 1 | **2** | 2 | 0 | **156** | 6 | 0 | 0 | **0** | 0 | 0 | 0 | 0 | 0 | **0** | **0** | 0 | 0 | 0 |
| N.t75 | **0** | 0 | **0** | 0 | 0 | 0 | **1** | 1 | 0 | **~800** | 0 | 0 | 0 | **1** | 0 | 0 | 0 | 0 | 0 | **0** | **0** | 0 | 0 | 0 |
| N.t76 | **0** | 0 | **0** | 0 | 0 | 0 | **2** | 2 | 0 | **46** | 6 | 0 | 0 | **1** | 0 | 0 | 0 | 0 | 0 | **0** | **0** | 0 | 0 | 0 |
| N.t77 | **0** | 0 | **3** | 0 | 0 | 3 | **0** | 0 | 0 | **0** | 0 | 0 | 0 | **0** | 0 | 0 | 0 | 0 | 0 | **0** | **0** | 0 | 0 | 0 |
| N.t78 | **0** | 0 | **0** | 0 | 0 | 0 | **0** | 0 | 0 | **2** | 0 | 2 | 0 | **0** | 0 | 0 | 0 | 0 | 0 | **0** | **3** | 3 | 0 | 0 |
| N.t79 | **0** | 0 | **0** | 0 | 0 | 0 | **1** | 1 | 0 | **0** | 0 | 0 | 0 | **0** | 0 | 0 | 0 | 0 | 0 | **0** | **0** | 0 | 0 | 0 |
| N.t80 | **0** | 0 | **0** | 0 | 0 | 0 | **1** | 1 | 0 | **~1000** | 30 | 0 | 0 | **0** | 0 | 0 | 0 | 0 | 0 | **0** | **1** | 0 | 0 | 1 |
| N.t81 | **0** | 0 | **0** | 0 | 0 | 0 | **0** | 0 | 0 | **26** | 2 | 0 | 0 | **0** | 0 | 0 | 0 | 0 | 0 | **0** | **0** | 0 | 0 | 0 |
| N.t82 | **0** | 0 | **2** | 0 | 0 | 2 | **1** | 1 | 0 | **0** | 0 | 0 | 0 | **0** | 0 | 0 | 0 | 0 | 0 | **0** | **0** | 0 | 0 | 0 |
| N.t83 | **0** | 0 | **2** | 0 | 0 | 2 | **1** | 1 | 0 | **0** | 0 | 0 | 0 | **0** | 0 | 0 | 0 | 0 | 0 | **0** | **1** | 1 | 0 | 0 |
| N.t84 | **0** | 0 | **0** | 0 | 0 | 0 | **1** | 1 | 0 | **~1500** | 0 | 0 | 0 | **0** | 0 | 0 | 0 | 0 | 0 | **0** | **0** | 0 | 0 | 0 |
| N.t85 | **0** | 0 | **3** | 0 | 0 | 3 | **1** | 1 | 0 | **0** | 0 | 0 | 0 | **0** | 0 | 0 | 0 | 0 | 0 | **0** | **0** | 0 | 0 | 0 |
| N.t86 | **0** | 0 | **8** | 0 | 0 | 8 | **1** | 1 | 0 | **2** | 1 | 0 | 0 | **0** | 0 | 0 | 0 | 0 | 0 | **0** | **0** | 0 | 0 | 0 |
| N.t87 | **0** | 0 | **16** | 0 | 0 | 16 | **0** | 0 | 0 | **5** | 4 | 0 | 0 | **0** | 0 | 0 | 0 | 0 | 0 | **0** | **0** | 0 | 0 | 0 |
| N.t88 | **0** | 0 | **36** | 0 | 0 | 36 | **0** | 0 | 0 | **420** | 420 | 0 | 0 | **0** | 0 | 0 | 0 | 0 | 0 | **0** | **0** | 0 | 0 | 0 |
| N.t89 | **0** | 0 | **0** | 0 | 0 | 0 | **1** | 1 | 0 | **4** | 4 | 0 | 0 | **0** | 0 | 0 | 0 | 0 | 0 | **0** | **2** | 2 | 0 | 0 |
| N.t90 | **0** | 0 | **0** | 0 | 0 | 0 | **0** | 0 | 0 | **1** | 1 | 0 | 0 | **1** | 0 | 0 | 1 | 0 | 0 | **0** | **0** | 0 | 0 | 0 |
| N.t91 | **0** | 0 | **1** | 0 | 0 | 1 | **3** | 3 | 0 | **22** | 11 | 0 | 0 | **2** | 0 | 0 | 0 | 0 | 0 | **1** | **0** | 0 | 0 | 0 |
