# Supplementary material for: Mesopredatory fishes from the subtropical upwelling region off NW-Africa characterised by their parasite fauna
Source: PeerJ. 2018 Aug 8;6:e5339. doi: 10.7717/peerj.5339 (PMC6087424; doi:10.7717/peerj.5339)
Supplement: Data S4 [file peerj-06-5339-s006.docx]

Supplemental Raw Data S4: Parasite specimens from *Trichiurus lepturus* deposited in the scientific collection of the Senckenberg Research Institute, Frankfurt am Main, Germany.

| Group | Species | Catalogue Nr. SMF | Preservation | Developmental stage | Host | Sampling region | Research cruise | Haul | Coordinates | Catch date | Identification | Original code | Collector |
| --- | --- | --- | --- | --- | --- | --- | --- | --- | --- | --- | --- | --- | --- |
| Digenea | *Lecithochirium microstomum* Chandler, 1935 | 15195 | Formalin & glycerine | Adult | *Trichiurus lepturus* | Canary Current System | Walther Herwig III 375. | 550/49 | N16°45.49', W16°38.16 | 29.06.14 | K. G. Alt | T.l39MD | K. G. Alt |
| Monogenea | *Octoplectanocotyla travassosi* Carvalho & Luque, 2012 | 15196 | Formalin & glycerine | Adult | *Trichiurus lepturus* | Canary Current System | Walther Herwig III 375. | 550/49 | N16°45.49', W16°38.16 | 29.06.14 | K. G. Alt | T.l27KM | K. G. Alt |
